# A clustering-based trajectory analytics of functional loss and recovery among older adults

**DOI:** 10.1371/journal.pone.0342424

**Published:** 2026-05-27

**Authors:** Ghazal Khalili, Manaf Zargoush, Somayeh Ghazalbash, Kai Huang

**Affiliations:** DeGroote School of Business, McMaster University, Hamilton, Ontario, Canada; Institut National d'Etudes Demograpiques: INED, FRANCE

## Abstract

**Objectives:**

Functional loss and recovery in older adults are heterogeneous, with important implications for independence, care needs, and survival. While Activities of Daily Living (ADLs) are routinely assessed, most existing approaches reduce them to summary scores, thereby losing information about the order, timing, duration, and recurrence of functional change. We introduce a novel trajectory analytics framework designed to identify clinically interpretable trajectory phenotypes and evaluate their association with mortality.

**Materials and methods:**

We analyzed 1.3 million ADL assessments from 265,530 residents in U.S. Veterans Affairs nursing homes. A hybrid trajectory clustering framework was developed, combining spell-based sequence construction, optimal-matching-based dissimilarity, and scalable quality-guided clustering. Sequence comparison was designed to preserve temporal structure while remaining computationally feasible for large-scale longitudinal data. Candidate clustering solutions were evaluated using multiple quality metrics, and mortality differences across clusters were examined using Kaplan-Meier estimation and Cox proportional hazards models adjusted for age and sex.

**Results:**

Thirteen distinct ADL trajectory clusters were identified from about 110,000 unique trajectories, differing in dominant disability states, duration, recurrence, and mortality risk. Short, severe trajectories showed the highest early mortality, whereas longer, milder trajectories involving limited impairments, such as walking or bathing, were more stable and had lower mortality. The highest-risk cluster showed approximately 48% cumulative mortality within the first year and remained associated with the greatest hazard of death after adjustment. Sex-based sensitivity analyses showed broadly similar mortality ordering across males and females, although female-specific estimates were less precise because of the smaller sample size.

**Discussion and conclusion:**

The proposed framework reveals substantial heterogeneity in functional trajectories and their prognostic implications, providing an interpretable and computationally efficient tool for large-scale ADL trajectory clustering. These findings support trajectory-based phenotyping as a useful approach for personalized care planning, targeted resource allocation, and policymaking in long-term care settings.

## Introduction

Functional mobility, referring to the ability to perform essential tasks required for independent living, is a critical determinant of quality of life, particularly in older adults. It has been referred to as the “sixth vital sign” in geriatric care [1,2], reflecting its role as a composite indicator of physical, psychological, and social well-being. Functional status is most commonly evaluated through Activities of Daily Living (ADLs), which encompass basic self-care and mobility tasks such as bathing, grooming, dressing, feeding, walking, transferring, toilet use, bowel continence, and urinary continence [3–6]. Although aging is often associated with functional decline, changes in ADL status are not necessarily linear, one-directional, or irreversible, and may include partial recovery, stabilization, or fluctuation over time. Individuals may experience periods of impairment followed by partial or full recovery, influenced by factors such as interventions, comorbidities, or lifestyle choices.

Loss of these abilities can negatively impact an individual’s safety, independence, and mental health [7,8]. ADLs are of greater importance in geriatrics, as aging is a major factor in the decline of these abilities [9–11]. In individuals aged 65 years and older, the lifetime risk of developing at least two ADL disabilities exceeds 70% [12], highlighting the importance of monitoring and understanding functional mobility changes in this population. The progression of functional impairment in older adults is often heterogeneous and temporal. Rather than a steady, irreversible deterioration, individuals may experience fluctuating patterns, including episodes of decline interspersed with recovery or stabilization. The temporal patterns of these changes, referred to as functional trajectories, offer valuable insights into prognosis, care needs, and potential intervention opportunities.

Several studies have explored ADLs trajectories using diverse methodologies, including machine learning techniques [11,13–22], typically focusing on summary scores or overall trends [15,19]. A few have analyzed the actual sequence of loss and recovery of ADLs [14], despite evidence that the order, likelihood, and timing of these events carry important clinical implications. Moreover, these analyses often rely on summary measures or aggregate trends and overlook the bidirectional nature of functional trajectories, in which recovery is possible, and trajectories may not follow a purely declining path.

Analyzing ADL trajectories on an individual level is very challenging. The number of possible ADL state sequences is combinatorially large, making direct case-by-case comparison impractical. Clustering provides an effective solution by grouping similar trajectories, facilitating a more organized and interpretable analysis. Rather than investigating a vast array of individual cases, this method enables clinicians to focus on a smaller selection of representative clusters, thereby providing clearer insights into functional health transitions and their implications. Importantly, no study has examined the trajectories of functional loss and recovery using a cluster-based trajectory analytics approach. This approach provides valuable insights into heterogeneous groups of trajectories, including transient and recoverable ones, helping to understand their distinct characteristics [21]. Given the dynamic and diverse nature of ADL disabilities, no single ADL pathway can represent all individuals [23]. Understanding functional trajectories is only meaningful if these patterns can be linked to outcomes that matter for patients, families, and healthcare systems. Without such connections, trajectory clusters remain descriptive rather than actionable.

Outcomes provide the basis for evaluating whether distinct trajectory patterns have meaningful prognostic and policy implications. Among possible outcomes, mortality is the most definitive and clinically consequential. As a clear and objective endpoint, death reflects the cumulative impact of functional disability, underlying illness burden, and comorbidity. Assessing variation in mortality risk across trajectory clusters, therefore, allows us to determine whether the identified patterns correspond to clinically distinct pathways rather than merely statistical groupings. More specifically, it helps differentiate trajectories that may be transient or partially reversible from those associated with sustained deterioration or terminal decline. This distinction has important implications for calibrating care intensity, identifying residents who may benefit from rehabilitation or palliative support, and anticipating future resource needs. By examining mortality as an outcome, our study strengthens the clinical relevance of trajectory clustering and highlights its potential value for both care planning and health system decision-making.

This study aims to address these knowledge and methodological gaps in analyzing ADL trajectories by introducing a novel hybrid clustering framework that combines Markov–based modeling with distance-based clustering algorithms. Markov models efficiently capture the probabilistic transitions between functional states, while distance-based methods offer detailed grouping of sequence patterns. The existing literature has utilized various methodologies to cluster diverse trajectory types, including finite mixture models, Markov models (MM), sequence analysis (SA), and distance-based algorithms (e.g., k-medoids, k-means, and hierarchical clustering) [24–36]. Our proposed hybrid approach overcomes computational limitations inherent in traditional sequence analytics tools, which struggle with the high dimensionality of large-scale longitudinal data. Applying this framework to over one million ADL assessments from U.S. Veterans Affairs nursing home residents, we aim to:

1. Identify distinct and clinically interpretable clusters of ADL trajectories,
2. Characterize their demographic, temporal, and functional features, and
3. Quantify differences in mortality risk across clusters.

By producing robust, scalable, and interpretable trajectory clusters, our approach offers a pathway toward ADL trajectory analytics, supporting personalized care planning, optimizing resource allocation, and informing health policy for aging populations.

## Materials and methods

### Data source and study population

To demonstrate the feasibility, scalability, and clinical interpretability of our proposed method for analyzing heterogeneous ADL trajectories, we used a large-scale longitudinal dataset from the VA Data Warehouse, processed on the VA Computing Infrastructure. This dataset encompassed information from the VA’s Electronic Medical Record System and nationwide Minimum Data Set (MDS) assessments performed in VA nursing homes, known as Community Living Centers (CLCs). As a standardized and compulsory clinical assessment tool, MDS provides comprehensive evaluations across multiple health domains. Collected by trained personnel, this information is sent to a central database for quality assurance and resource management purposes. This dataset consists of de-identified records that are publicly accessible, previously published [14,17,18], and compliant with data-sharing guidelines. Information on data access and the accompanying data dictionary is provided in the Data Availability Statement and the project repository.

The dataset comprises 1,328,052 ADL assessment records collected from 265,530 residents in CLCs between January 1, 2000, and October 9, 2012. Each resident underwent numerous assessments, enabling effective tracking of their individual ADL trajectories over time. The assessments capture nine critical physiological functions: bathing (denoted by B), grooming (G), dressing (D), feeding (F), transferring (S), walking (W), toilet use (T), bowel continence (L), and urinary continence (U). Additional demographic variables include age, sex, assessment time (from first visit), and mortality status. Among 265,523 residents with valid age information, the average age at the initial assessment was 71.07 years (Standard Deviation, SD = 12.26), and the population was predominantly male (96.9%). After applying exclusion criteria (removing individuals with no follow-up, invalid age values, inconsistent death ordering, and individuals with insufficient longitudinal information), the dataset was refined to include 846,859 valid assessment records, representing 148,750 patients. ADL assessments were recorded as part of routine care rather than at fixed intervals. Therefore, trajectories were constructed based on the chronological sequence of observed assessments without imputing or interpolating unobserved time points. The median number of assessments per patient was 6 (IQR: 4–9), while the mean was 8.1 (SD: 7.3), with a range from 3 to 107 assessments. The median follow-up duration was 1.95 years (IQR: 0.63–4.17), corresponding to a mean of 2.77 years (SD: 2.66) and with follow-up extending up to 14.6 years. These statistics demonstrate considerable longitudinal depth and heterogeneity in observation time across patients, providing a robust foundation for trajectory construction and clustering. For detailed information on the dataset, please refer to [18,22].

### Trajectory extraction

To enable trajectory analytics, we used binary coding to transform the original multi-domain ADL disability data into a compact, state-based representation that captures the presence or absence of disability. Each ADL assessment includes nine binary-coded functional domains: B, G, D, F, S, W, T, L, and U. For each assessment, we encoded the patient’s functional status as a single “state” in a unique combination of impaired domains. For example, a patient unable to groom, bathe, and walk would be represented by the state GBW, assigned the numeric code “52.” In our study, the state labels GBW, GWB, and WBG all represent the same underlying disability combination (impairments in grooming, bathing, and walking) and are therefore treated as the same disability states. What distinguishes trajectories, however, is the order in which patients move into and out of such states. Our trajectory analytics explicitly capture this temporal sequencing, ensuring that while equivalent states are grouped consistently, the pathways leading to them remain preserved. The nine ADL domains permit a large number of possible disability combinations, but the empirical distribution of observed states was highly skewed. The 25 most frequent states accounted for approximately 86% of all assessments, whereas the remaining states were individually rare and collectively represented a highly sparse portion of the state space. To reduce sparsity and computational burden while preserving the dominant recurring disability patterns, and consistent with prior research [22], we restricted the clustering analysis to these 25 most frequent states. They are listed in Table S1 in S2 Appendix, alongside their associated numeric codes.

We utilized the TraMineR package in R [37] to transform the assessment records into state sequences. The input data were structured in SPELL (state-duration) format, with each row representing a time interval during which an individual remains in a specific state, defined by the start and end day of that interval. In this representation, it is assumed that the observed state remains unchanged between two consecutive assessments. Consequently, the sequences were recorded as successive (state, duration) spells rather than being expanded into daily observations. The maximum duration of assessment in our dataset is 5331 days, which determines the overall temporal range of the trajectories. Individual sequences vary in length depending on each patient’s observation period.

Because the dataset contains 148,750 patient-level trajectories, sequence comparison becomes computationally intensive. To address scalability challenges, we merged identical trajectories and assigned frequency weights [38] corresponding to the number of patients sharing each exact sequence, resulting in 109,956 unique trajectory patterns.

### Trajectory clustering

**Fig 1:**
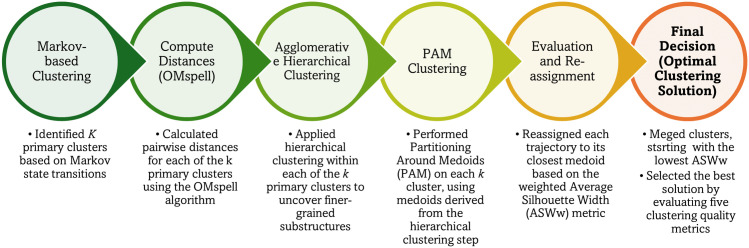
Pipeline of the Proposed Framework.

Trajectory clustering is a process of grouping trajectories into clusters with distinct profiles. Broadly, sequence clustering methods can be divided into two families: distance-based approaches, which compute pairwise dissimilarities between sequences and model-based approaches, such as those utilizing Markovian models, which estimate the probability structure underlying sequence transitions [34]. Distance-based methods are widely used in the literature for their flexibility and interpretability. However, they become computationally prohibitive for large datasets, as calculating all pairwise distances increases quadratically with the number of sequences. Model-based approaches are computationally more efficient but may sacrifice granularity in capturing structural variations. To leverage the strengths of both, we propose an integrated hybrid approach that incorporates both clustering approaches. Our algorithm consists of five steps, as depicted in Fig 1.

**Phase 1: Markov-based Clustering**: The data set is first analyzed using Markov-based clustering to form primary clusters based on the transition probabilities between different states in the trajectories [38]. The algorithm includes four steps, starting with a random initialization of the transition matrices for *k* clusters. In the next step, known as the expectation (E) step, we estimate the likelihood that each sequence was generated by each cluster *c*_*k*_. To do this, we employ a Markov chain model, where the probability of a trajectory is determined by the product of the transition probabilities $P(x_{i}|x_{i-1};c_{k})$, which indicates the probability of transitioning from state *x*_*i*−1_ to state *x*_*i*_ within cluster *c*_*k*_ (Equation 1). Subsequently, each trajectory is allocated to the cluster with the highest probability. In the third step, namely the maximization (M) step, the transition matrices are updated for all clusters. Finally, the E and M steps are repeated iteratively until both the transition matrices and sequence assignments converge (i.e., minimal change between iterations).


$$\begin{array}{c} P(x|c_{k})=\prod_{i=1}^{a+1}P(x_{i}|x_{i-1};c_{k}) \end{array}$$ (1)

This phase produces a small number of primary clusters that capture overall variations in trajectory patterns while drastically reducing the number of pairwise comparisons required in the subsequent phase.

**Phase 2: Distance-based Clustering**: We perform a two-step distance-based refinement for each primary cluster from Phase 1:

1. **Agglomerative Hierarchical Clustering:** We first compute pairwise dissimilarities among sequences within a primary cluster and apply agglomerative hierarchical clustering using the average linkage criterion. This criterion balances cluster compactness and separation [39], making it well-suited to high-dimensional sequence data.
2. **Partitioning Around Medoids (PAM):** The medoids from the hierarchical solution serve as initialization points for PAM [40]. PAM iteratively swaps medoids with non-medoid sequences to minimize within-cluster dissimilarity, reducing sensitivity to random initialization and improving stability.

**Measuring Dissimilarities**: We use the OMspell variant of the Optimal Matching (OM) algorithm [41], which extends standard OM by comparing “spells” (continuous runs of the same state). OMspell captures three critical sequence dimensions, i.e., order (sequencing), timing, and duration, and allows for parameter tuning to adjust sensitivity to each of these dimensions. OM accommodates timing shifts and unequal sequence lengths through substitution, insertion, and deletion operations during distance computation. This flexibility ensures that the clustering process can emphasize, for example, whether differences in the order of events, the length of time spent in particular states, or the exact timing of transitions are most relevant to distinguishing trajectories. Trajectories were anchored at each resident’s first eligible ADL assessment and ended at the last observed assessment. Sequences were not artificially extended or imputed beyond observed follow-up. Substitution costs are determined from state attributes (i.e., severity levels of disability combinations), reflecting clinical similarity between states. A constant INDEL (insertion/deletion) cost is used for simplicity, following Equation S1 [41] in S4 Appendix. Determining the Optimal Number of Clusters: For each primary cluster, we run PAM with *k* and evaluate solutions using the weighted average silhouette width (ASWw) [38]. The ASWw accounts for sequence weights and measures how well each sequence fits within its cluster compared to its nearest alternative. The k yielding the highest ASWw is selected. More details of Step 2 are available in S4 Appendix.

**Phase 3: Evaluation and Optimization** The goal of Phase 3 is to ensure that the final clustering solution is both computationally tractable and clinically meaningful, balancing statistical rigor with interpretability of the resulting trajectory groups.

**Step 3.1: Global Reassignment** After Phase 2, each primary cluster contains several refined sub-clusters. These are pooled together into a global candidate set of medoids (one medoid per sub-cluster). Each sequence is then reassigned to the nearest medoid across all candidates, regardless of its Phase 1 cluster of origin. This reassignment is based on OMspell distance (S4 Appendix, Phase 2) and accounts for the fact that some sequences in different primary clusters may be more similar to each other than to sequences within their original group. Computationally, this reduces the distance matrix size from *N* × *N* (where *N* = 109,956) to *N* × *m* (where *m* is the number of candidate medoids), which dramatically decreases memory and runtime demands while retaining the full set of trajectory–cluster relationships. Clinically, this step ensures that the clustering reflects true functional trajectory similarity rather than artifacts of the initial Markov grouping.

**Step 3.2: Quality-Driven Iterative Merging** While the global reassignment yields an initial comprehensive clustering solution, some clusters may be poorly defined (e.g., containing only marginally distinct sequences or representing noisy outliers). To refine the solution, we use a quality-driven merging process:

1. Evaluate cluster quality using five complementary metrics:
  - **Hubert’s Somers’ D (HGSD):** Measures the agreement between the original dissimilarity matrix and the one implied by the clustering [42]. Higher values indicate more faithful reconstruction of the original distances.
  - **Hubert’s C Index (HC):** Compares the clustering against an idealized “best possible” solution [42]. Lower values are better.
  - **Calinski–Harabasz Index (CH):** Balances cluster compactness and separation using an ANOVA-like ratio [43]. Higher values indicate better-defined clusters.
  - **Pseudo-*R*^2^:** Represents the proportion of variance in sequence dissimilarities explained by the clustering [44].
  - **Weighted Average Silhouette Width (ASWw):** Captures how well each sequence fits into its cluster compared to the nearest alternative, accounting for sequence weights [38].
2. Identify the lowest-performing cluster according to ASWw (our primary optimization criterion, as it reflects within-cluster cohesion vs. between-cluster separation).
3. Merge the weak cluster with its most similar neighbor, determined by the smallest average OMspell distance between medoids.
4. Recalculate medoids for any affected clusters and reassign sequences accordingly.
5. Repeat Steps 1–4 until the merging process reaches an optimal trade-off between cluster compactness, separation, and interpretability.

**Step 3.3: Selecting the Optimal Number of Clusters** Instead of fixing k in advance, our approach lets the number of clusters emerge from the optimization process. We monitor all five metrics during iterative merging and select the configuration that achieves: high ASWw and CH values; high HGSD and pseudo-R2; low HC; and a manageable number of clusters for clinical interpretability (avoiding dozens of micro-clusters that are statistically different but not operationally actionable).

Full details of Phase 3 are in S4 Appendix.

### Mortality analysis

To examine whether the identified ADL trajectory clusters differed in mortality risk, we conducted time-to-event analyses using all-cause mortality as the endpoint. Baseline was defined as the date of each resident’s first eligible ADL assessment included in the analytic cohort, and follow-up time was measured from baseline to the earliest of recorded death or the end of the last observed assessment interval. Residents without a recorded death were treated as right-censored at their last observed follow-up date. We first estimated cluster-specific survival functions using the Kaplan-Meier method to compare survival patterns over time across trajectory groups. We then fitted Cox proportional hazards regression models to quantify the association between cluster membership and mortality risk while adjusting for baseline age and sex as potential confounders. Hazard ratios and 95% confidence intervals were estimated using Cluster #1 as the reference group. Because the available dataset did not include alternative terminal outcomes or sufficiently reliable competing-event information, we did not implement a competing-risks model; accordingly, death was treated as the sole event of interest, and discontinuation of follow-up without recorded death was handled as right-censoring under the standard non-informative censoring assumption.

All analyses were conducted using R (version 4.1). Sequence construction and dissimilarity computation were performed using the TraMineR package (version 2.2–7). To facilitate reproducibility, a detailed summary of key parameter settings is provided in Table S3 of the S2 Appendix.

## Results

Our hybrid clustering framework successfully processed over one million ADL assessments from 265,530 residents, producing clinically interpretable functional trajectory clusters at a scale that would be computationally unfeasible with traditional sequence analysis methods. The results demonstrate both the methodological feasibility of our approach and its clinical value in clustering older adults according to distinct patterns of functional decline and recovery.

### Markov-based clustering

The initial Markov-based stage produced four primary trajectory clusters (*k* = 4), serving as a partitioning that dramatically reduced computational demands for subsequent distance-based refinement. Convergence was reached after eight iterations, with minimal changes in trajectory assignments (< 3.5%) and stable transition matrices (mean pointwise difference = 0.07). Table 1 illustrates the distribution of trajectories across the four clusters derived from the Markov chain analysis. We note that cluster #1 comprises a significant proportion of the trajectories, whereas the number of sequences in the remaining three clusters is relatively similar, indicating a more balanced distribution among these clusters.

**Table 1:** Counts of trajectories in each cluster in phase 1.

Cluster	#1	#2	#3	#4
Count	60437	15798	15410	18311
Percentage	55	14	14	17

**Fig 2:**
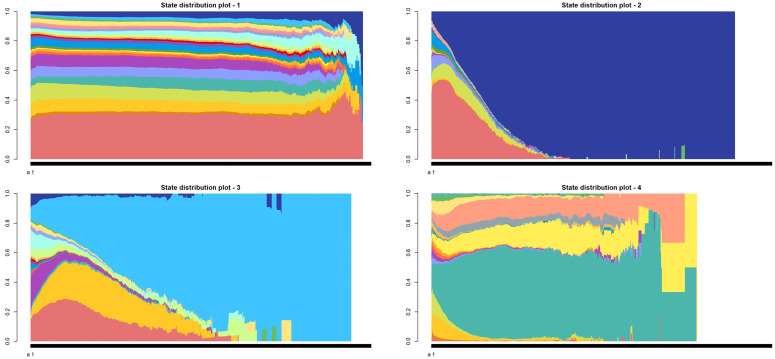
State distribution plots of clusters in Phase 1. Note: Each color represents a distinct state. The full color legend is provided in S3 Appendix, Figure S3.

Fig 2 illustrates the state distribution across the four clusters. The first cluster closely mirrors the overall data distribution, whereas the remaining clusters display distinct patterns. Cluster #2 reveals a significant discrepancy where patients exhibit all impairments (“All”) by the end of the assessment. In cluster #3, state SGTBDWLU shows a higher probability of occurrence, particularly increasing toward the end of the observation period. Lastly, cluster #4 features a prominent combination of disability W.

### Distance-based clustering (Integration of Hierarchical & PAM Clustering)

**Fig 3:**
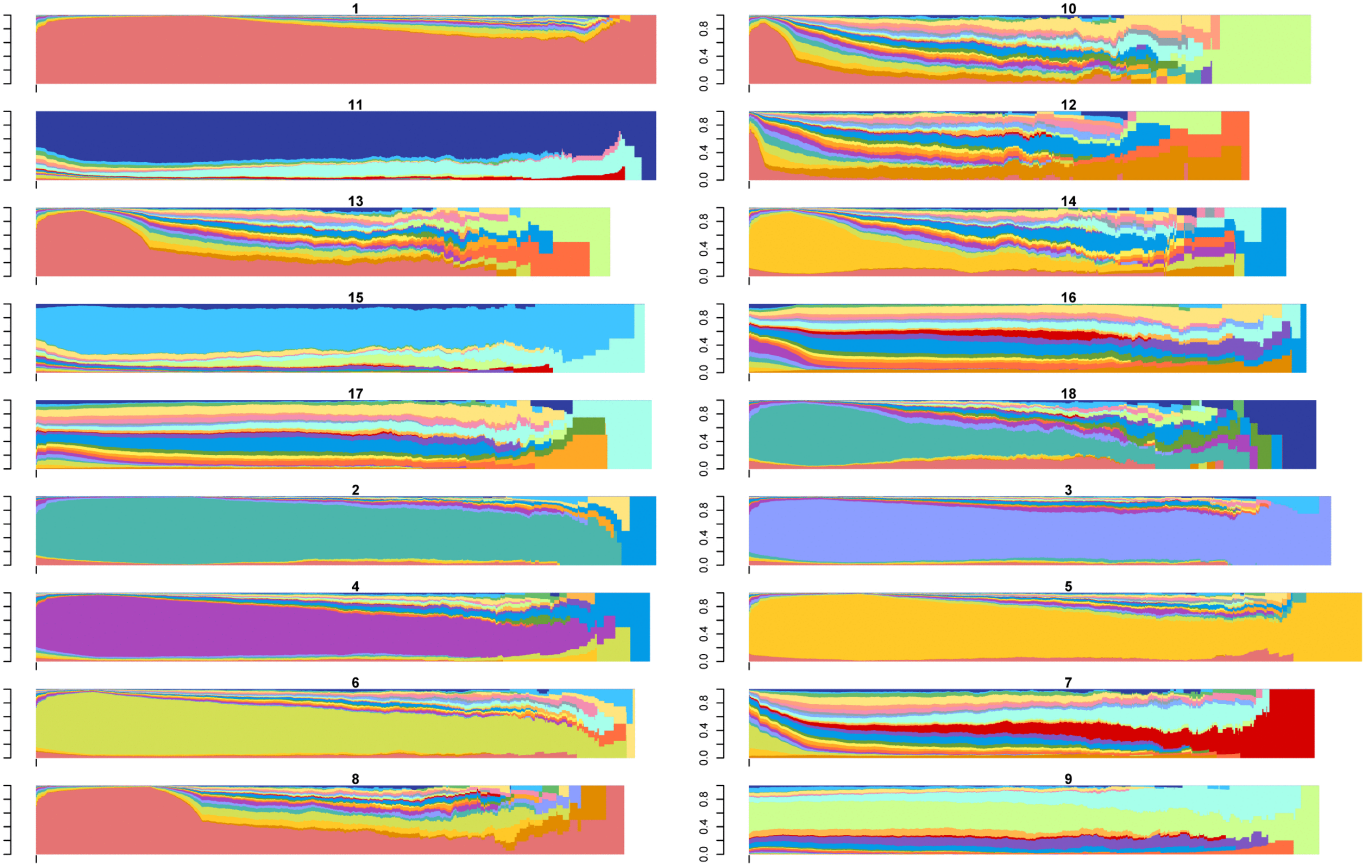
State distribution of 18 clusters initially obtained in phase 2. Note: Each color represents a distinct state. The full color legend is provided in S3 Appendix, Figure S3.

Using the output from this hierarchical clustering as the initial medoids, we then implemented the PAM algorithm with a range of cluster counts (k∈{2,3,…,20}). Subsequently, we evaluated the quality of each clustering solution using the weighted ASW metric and selected the optimal value of k based on the highest ASWw score. As a result of this process, we created a total of 18 sub-clusters, with clusters #1, #2, #3, and #4 yielding 9, 3, 4, and 2 sub-clusters, respectively. The initially obtained sub-clusters are visualized in Figures S4-S7 S3 Appendix. The combined distance-based algorithms were applied independently within each primary cluster to create these sub-clusters. Therefore, in the next step, we calculated the distances between the medoids of these 18 sub-clusters and each trajectory. Afterward, we reassigned each trajectory to the nearest medoid, which may differ from its original assignment. This step highlights one advantage of our algorithm: computing a smaller distance matrix with dimensions [109,956 × 18] instead of a much larger matrix sized [109,956 × 109,956]. Reducing the size of the distance matrix was essential for overcoming the limitations imposed by the current state of SA tools and for making the clustering process computationally feasible. Fig 3 illustrates the state distribution plots of the 18 clusters, providing a comprehensive overview of the prominent states within each cluster.

The resulting sub-clusters revealed critical differences in disability composition, trajectory duration, and transition patterns. For example, within the “walking-disability” group, sub-clusters varied in whether walking disability was preceded by broader multi-domain impairments or occurred in isolation.

### Evaluation and optimization

**Fig 4:**
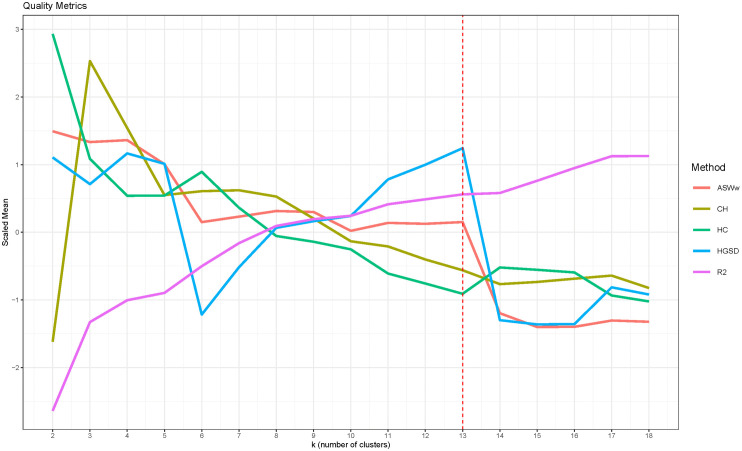
Quality assessment of the clustering settings.

We applied the quality assessment procedure to iteratively refine the initial clustering solution. During this process, clusters were merged successively based on their evaluation scores, following this order: 17⇝7⇝9⇝3⇝16⇝15⇝1⇝11⇝13⇝14⇝4⇝6⇝10⇝18⇝5⇝8. Figure S8 in S3 Appendix illustrate how the state distribution changed after each merge. The clustering quality was assessed using five established metrics, and the results indicated that the optimal number of clusters was k^*^ = 13, as this setting provided the best overall balance across all metrics (Fig 4).

**Fig 5:**
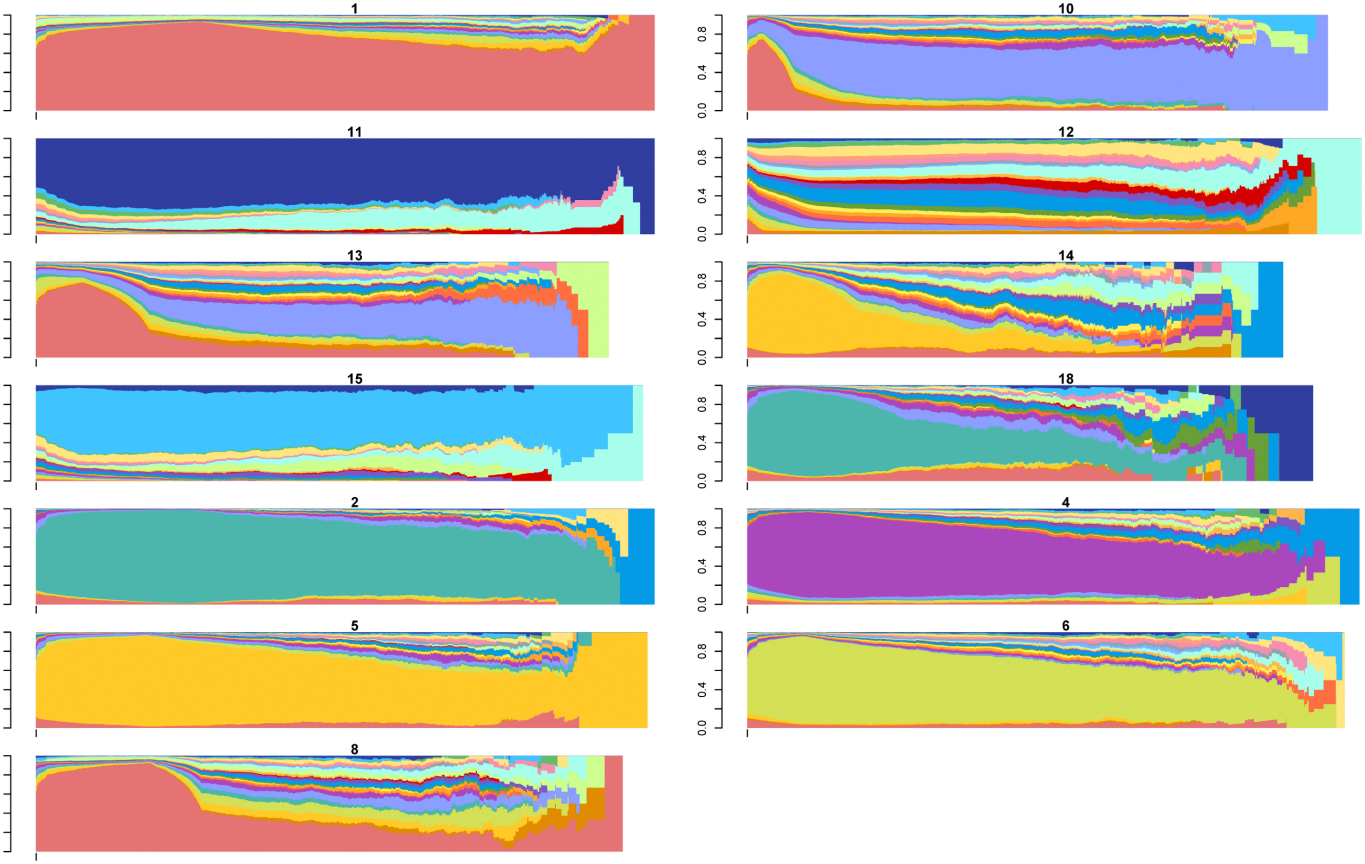
State distribution of the optimal clustering setting (*k* = 13).

Fig 5 shows the state distribution of the 13 final clusters, which differ in their dominant combinations of disabilities (indicated by color) and the duration spent in each disability state. This highlights the distinct characteristics of each cluster. Additionally, Figure S9 in S3 Appendix presents the relative frequency for each final trajectory cluster. These plots illustrate the medoids of 250 equally sized sequential trajectory groups within each cluster, providing a detailed view of functional loss and recovery patterns over time.

### Representative trajectories

We selected representative trajectories based on four quality dimensions, which include density, centrality, frequency, and likelihood. We then compared the quality of the sets produced by each method. To ensure meaningful coverage of each cluster while maintaining interpretability, we established a minimum coverage threshold of 75%, which effectively balances representativeness and the set size. At the same time, we limited the number of representative trajectories to a maximum of 10 per cluster to keep the results concise and manageable for interpretation. These thresholds represent a practical balance between analytical depth and clarity, particularly when presenting complex trajectory data. Among the sets that met both conditions, we chose those with the optimal combined performance regarding coverage, number of representatives, and gain value. This approach enables us to identify representative sets that effectively capture the typical characteristics of the clusters while maintaining a balance between coverage, size, and the quality gained. The results of this analysis are presented in Table S2, S2 Appendix. Overall, the centrality and likelihood criteria produced higher-quality representative sets for most clusters. An exception was cluster #6, where the frequency-based method yielded better results than the others. Therefore, we prioritized centrality and likelihood over density and frequency to maintain consistency and interpretability. These findings highlight the importance of considering multiple criteria for trajectory clustering rather than relying solely on a single criterion. Trajectories representing each cluster are presented in Table 2, and illustrated in Figure S10, S3 Appendix.

**Table 2:** Representative set of final clusters.

Cluster #1	Cluster #2	Cluster #4	Cluster #5	Cluster #6
**[1]** (0,1396) **[2]** (0,2388) **[3]** (0,3380) **[4]** (0,4421)	**[1]** (32,1318) **[2]** (32,2156) **[3]** (32,3001)	**[1]** (52,519) **[2]** (52,1364) **[3]** (52,2213) **[4]** (52,3088)	**[1]** (16,1444) **[2]** (20,18) (16,2393)	**[1]** (20,490) **[2]** (20,1345) **[3]** (20,2500)
Cluster #8	Cluster #10	Cluster #11	Cluster #12	Cluster #13
**[1]** (0,1126)	**[1]** (0,213)	**[1]** (511,375) **[2]** (511,1289) **[3]** (511,2205) **[4]** (511,3141) **[5]** (511,4069) **[6]** (511,1857)-(255,661) **[7]** (511,2785)-(255,699) **[8]** (511,3359)-(127,1583) **[9]** (511,804)-(255,726) **[10]** (511,2469)-(52,844)	**[1]** (52,2)	**[1]** (0,549) **[2]** (20,279) (16,121)
Cluster #14	Cluster #15	Cluster #18		
**[1]** (16,542)	**[1]** (510,347) **[2]** (510,1257) **[3]** (510,2177) **[4]** (510,3264) **[5]** (510,5021) **[6]** (508,794)-(510,517) **[7]** (510,1558)-(16,362)-(0,726) (20,410)-(0,571)-(254,11) **[8]** (510,1784)-(511,568) **[9]** (52,570)-(510,858) **[10]** (510,765)-(511,645)	**[1]** (32,394) **[2]** (32,1134)		

### Final cluster characteristics

Table 3 and Fig 5 provide valuable insights into the structural characteristics of the final clusters, including cluster proportions, percentages of male residents, average ages, average trajectory lengths, average numbers of visited states, and average recurrence degrees. Fig 5 illustrates the temporal distribution of disability states across each cluster. The x-axis represents the follow-up time since the initial ADL assessment, while the y-axis shows the proportion of residents in each disability state at specific time points. Each colored band indicates the share of individuals in that cluster experiencing a particular combination of ADL impairments over time. The dominant color patterns within each panel, therefore, indicate the most common disability states characterizing that trajectory cluster. This visualization, in conjunction with the representative trajectories presented in Table 2, effectively demonstrates how functional states evolve across the clusters. Cluster descriptive titles were assigned based on the dominant and persistent disability states observed in Fig 5 and the overall trajectory patterns summarized in Table 2. For clarity, clusters are referenced by their numeric identifiers (as in Table 2), while descriptive labels summarize their dominant functional profiles (see Table 3). Overall, the clustering algorithm separated trajectories into distinct functional profiles characterized by differences in trajectory length and dominant disability states.

**Table 3:** Summary of characteristics of final clusters.

Cluster	% of Unique Trajectories	% of Male Residents	Ave. Age	Ave. Length	Ave. # of States	Ave. Rcurr.	Descriptive Title
1	6.39	97.0	65.86	2340	2.5	1.14	Stable no-disability
2	1.66	97.8	63.18	2406	2.3	1.11	Persistent walking impairment
4	3.49	97.4	71.52	1682	2.6	1.10	Persistent bathing-grooming impairment
5	2.69	95.8	72.17	1955	2.6	1.10	Persistent bathing impairment
6	4.53	96.6	75.14	1567	2.7	1.11	Persistent grooming-bathing impairment
8	3.42	97.2	67.41	1489	2.9	1.17	Progressive multi-domain (gradual)
10	7.17	97.1	68.43	806	2.9	1.16	Rapid progression to bathing-walking impairment
11	2.85	95.4	72.43	1570	2.7	1.22	Long-term full-dependency
12	52.22	97.0	71.82	434	2.4	1.08	Rapid full-dependency decline
13	8.21	97.2	69.33	1037	3.0	1.17	Progressive multi-domain (accelerated)
14	3.23	96.7	73.79	938	3.0	1.13	Fluctuating multi-domain
15	1.66	94.8	73.07	1553	3.0	1.21	Persistent severe multi-domain
18	2.48	98.0	65.63	1170	2.7	1.16	Late progressive walking-multi-domain decline

Although the cohort is predominantly male, Clusters #15 (persistent severe multi-domain) and #11 (long-term full-dependency) have relatively lower proportions of male residents compared with other clusters. These two clusters are also characterized by the most severe combinations of disabilities, specifically *SGTBWDLU* (state 510) and *All* (state 511), as shown in Fig 5 and Table 2. Correspondingly, both clusters exhibit the highest recurrence degrees (1.22 and 1.21, respectively; Table 3), indicating that residents frequently cycled through phases of recovery and decline throughout their disability trajectories. Such patterns may suggest more complex health conditions, potentially reflecting repeated hospitalizations or functional instability. Additionally, the recurrence degree negatively correlates with the proportion of male residents, suggesting that male residents may experience relatively more stable trajectories compared to females. Furthermore, Clusters #13 (progressive multi-domain), #14 (fluctuating multi-domain), and #15 have the highest average number of visited states (3.0), suggesting greater diversity in functional states over time.

In contrast, clusters with longer trajectory lengths, such as Cluster #1 (stable no-disability) and Cluster #2 (persistent walking impairment), which extend beyond six years on average, tend to involve fewer distinct states (2.3 and 2.5) and lower recurrence degrees (∼1.1), suggesting relatively stable functional trajectories in which residents remain in similar disability states for extended periods.

Cluster #2, dominated by the walking impairment state, includes the youngest residents on average, while Cluster #6 (persistent grooming–bathing impairment) includes the oldest residents. Across clusters, residents experiencing more severe or multi-domain disability states also tend to be older on average. Notably, Cluster #12 (rapid full-dependency decline) represents the largest proportion of trajectories (52.22%) and is characterized by very short trajectory durations (average length 434 days), suggesting rapid functional decline within a relatively short follow-up period.

Clusters also differ substantially in their overall trajectory durations. Clusters #1 and #2 exhibit the longest trajectories, each spanning more than six years, whereas Clusters #10 (rapid decline to bathing–walking impairment) and #12 have much shorter durations, averaging less than 2.5 years. While longer trajectories might intuitively involve more transitions between disability states, the observed patterns suggest the opposite: clusters with longer durations tend to involve fewer distinct states and lower recurrence, indicating greater persistence within particular disability states. In contrast, shorter trajectories tend to switch states more quickly, leading to more transitions despite their shorter duration. This indicates more dynamic or unstable functional states. As discussed in the following section, these structural differences in trajectory dynamics align closely with the mortality patterns observed across clusters.

A related descriptive pattern can also be observed between age and sex composition across clusters. Clusters with higher average ages, such as Clusters #6 (75.1 years), #14 (73.8 years), #15 (73.1 years), and #11 (72.4 years), tend to include slightly lower proportions of male residents (approximately 94–96%). In contrast, clusters with younger residents, including Clusters #2 (63.2 years), #1 (65.9 years), and #18 (65.6 years), have higher male proportions (around 97–98%). Although these differences are modest and the cohort remains predominantly male overall, the pattern suggests that female residents appear somewhat more frequently in clusters associated with older age and more severe functional disability.

### Mortality risk across clusters

**Fig 6:**
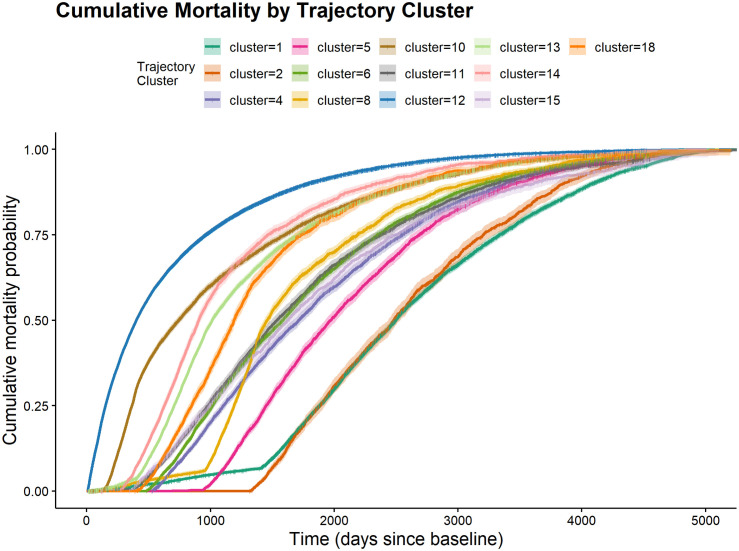
Kaplan-Meier estimates of cumulative mortality for different clusters.

Fig 6 presents Kaplan-Meier estimates of cumulative mortality across the 13 trajectory clusters, revealing substantial heterogeneity in survival patterns. Clusters #12 and #10 exhibited the highest early mortality, with cumulative mortality rates of approximately 48% and 26%, respectively, within the first year of follow-up. In contrast, several clusters, including #2, #4, #5, #6, and #18, showed essentially no mortality during the first year. Notably, Cluster #12 was not only the most prevalent trajectory pattern in the cohort but also the one with the highest early mortality, suggesting that it may represent a common terminal disability pathway.

The temporal pattern of mortality also differed meaningfully across clusters. Cluster #11 showed a delayed but pronounced increase in mortality: its early survival profile was comparable to that of several other clusters during the first several years of follow-up, but cumulative mortality increased sharply thereafter, exceeding 85% before nine years. In contrast, Clusters #1, #2, and #5 all exhibited relatively low early mortality but diverged in longer-term risk. Cluster #1, characterized primarily by no disability, maintained the lowest mortality overall, with approximately 22.8% cumulative mortality at five years. Cluster #2, characterized by persistent walking impairment, showed a delayed increase in mortality after approximately three years, whereas Cluster #5, marked by bathing impairment, demonstrated a clearer rise beginning around the third year and reached approximately 43.9% cumulative mortality by five years. These patterns indicate that clusters associated with milder or more stable disability states tend to have lower early mortality, whereas trajectories involving more persistent or progressive functional limitations are associated with worse longer-term survival. Finally, Clusters #4, #6, #8, and #18 displayed intermediate mortality patterns, with neither the extreme early mortality nor the delayed, sharp increase observed in Cluster #11. These clusters were associated with prominent states such as *GBW* (state 52), *GB* (state 20), *O* (state 0), and *W* (state 32), respectively, suggesting moderate levels of disability severity and mortality risk. Overall, the Kaplan-Meier curves support the clinical relevance of the trajectory solution by showing that distinct functional pathways are associated with markedly different survival profiles.

The age- and sex-adjusted Cox proportional hazards model yielded results consistent with the Kaplan-Meier analysis. Relative to Cluster #1 as the reference, Cluster #12 remained associated with the highest mortality risk (HR = 5.00, 95% CI: 4.86–5.15), followed by Cluster #10 (HR = 2.95, 95% CI: 2.84–3.06). Cluster #11, which exhibited delayed mortality in the Kaplan-Meier curves, also showed elevated risk (HR = 1.65, 95% CI: 1.57–1.72). Clusters characterized by milder disability trajectories showed smaller increases in mortality risk, including Cluster #2 (HR = 1.10, 95% CI: 1.04–1.17) and Cluster #5 (HR = 1.24, 95% CI: 1.18–1.30). Clusters with intermediate mortality patterns in the survival curves, including #4, #6, #8, and #18, showed moderately elevated hazards, ranging from 1.49 to 2.42. Together, these findings indicate that the mortality differences across trajectory clusters persist even after adjustment for demographic factors.

An important consideration when interpreting these findings is the demographic composition of the cohort, which is predominantly male (∼97%). This reflects the demographic structure of the VA CLC population and limits the direct generalizability to female-predominant or more sex-balanced long-term care settings. To evaluate the potential impact of this sex imbalance, we performed sensitivity analyses including sex-stratified Kaplan–Meier curves, sex-specific Cox proportional hazards models adjusted for age, and a model with cluster-by-sex interaction terms. Notably, the relative ordering of trajectory clusters by mortality risk remained highly consistent between males and females. For example, clusters with the highest hazards in the primary analysis, such as Cluster #12 (HR = 5.01, 95% CI 4.87–5.16) and Cluster #10 (HR = 2.96, 95% CI 2.85–3.07), showed similarly elevated risks. In the interaction model, we did not observe strong statistical evidence that the association between trajectory cluster and mortality differed by sex (all interaction *p*-values > 0.05). Moreover, the distribution of residents across trajectory clusters was remarkably similar between sexes. Cluster #12 was the most prevalent group in both males (52.27%) and females (50.32%), with other clusters showing closely comparable proportions (Table S4, S2 Appendix). Although estimates in females were less precise due to the substantially smaller sample size, resulting in wider confidence intervals (Figure S12, S3 Appendix), the direction and magnitude of cluster-specific associations with mortality were broadly similar to those observed in males. These findings suggest that the identified trajectory phenotypes and their prognostic associations with mortality are robust to the marked sex imbalance in the cohort and are unlikely to be driven solely by the male predominance of the cohort.

## Discussion

This study presents a novel, hybrid clustering framework for analyzing trajectories of functional loss and recovery among older adults, operationalized through ADLs. By integrating Markov chain–based sequence modeling with distance-based clustering, our approach overcomes major computational limitations that have historically restricted the scope of sequence analysis in large-scale clinical datasets. Applying this framework to more than one million ADL assessments from VA nursing homes, we identified 13 clinically interpretable trajectory phenotypes that differ substantially in severity, recurrence, demographic composition, and mortality risk.

Our analysis identified 13 clinically interpretable ADL trajectory phenotypes that capture meaningful heterogeneity in how functional limitations evolve over time among older adults in long-term care. These trajectories were not distinguished solely by disability severity, but also by their temporal structure, including stability, recurrence, progression, fluctuation, and concentration in severe dependency states. For example, some clusters reflected prolonged functional independence or relatively isolated impairment in a single ADL domain, whereas others reflected persistent multi-domain disability, progressive worsening, or highly unstable functional courses. These distinctions are clinically important because individuals with similar disability burden at a single time point may nevertheless follow very different longitudinal pathways, with different implications for care needs, prognosis, and intervention timing.

### Advancing the understanding of functional trajectories

Prior research on ADL trajectories has often relied on aggregate scores or limited trend categories [15,19], thereby overlooking the temporal order and pattern complexity of functional decline and recovery. While a few studies have examined sequences of functional change [14], these analyses have typically been constrained to small samples, single domains, or methods that cannot scale to large datasets. Our work advances the field by a) capturing the whole multi-domain temporal progression of functional states, including both disability progression and recovery events. b) using a two-phase clustering strategy that preserves fine-grained structural differences while remaining computationally feasible for nearly 110,000 unique sequences, c) linking trajectory phenotypes directly to mortality risk, thereby demonstrating their prognostic utility. The result is a data-driven taxonomy of functional trajectories that reveals heterogeneity masked by summary measures. For example, two individuals with identical baseline and end states may follow entirely different ADL paths with different mortality risks that would be indistinguishable in traditional models.

### Managerial and policy implications

The insights from our study offer numerous benefits and applications across society, healthcare systems, and decision-making processes. By identifying 13 distinct long-term ADL trajectory clusters, ranging from stable, no-disability and persistent single-domain impairments (e.g., walking- or bathing-impairment trajectories) to rapid full-dependency decline and fluctuating multi-domain disability trajectories, our analysis reveals substantial heterogeneity in functional decline patterns among long-term care residents.

First, predictive models could be developed to assign new residents to the most similar trajectory cluster early in their stay. This cluster membership could then serve as a prognostic tool to forecast likely ADL progression, time to severe dependency, and mortality risk. Early identification of trajectory membership may enable clinicians to intervene before irreversible functional decline occurs and tailor care strategies accordingly. For example, residents falling into the rapid full-dependency decline trajectory (Cluster #12) or the rapid decline to bathing–walking impairment trajectory (Cluster #10) experience very high early mortality and short functional survival. These trajectories may benefit from early palliative care consultation and strategies that prioritize supportive, comfort-focused care over aggressive medical interventions. In contrast, individuals in stable no-disability (Cluster #1) or persistent walking impairment trajectories (Cluster #2) exhibit prolonged independence or mild limitation with low early mortality. These clusters may benefit from proactive rehabilitation, fall-prevention programs, and mobility-preserving interventions, such as strength and balance training, to maintain functional independence [45]. Residents with fluctuating or persistent severe multi-domain disability trajectories (such as Clusters #14, #15, and #11) show high recurrence rates and often cycle through phases of recovery and decline. These patterns highlight the importance of frequent functional reassessment and early interventions to prevent further functional decline.

Second, cluster-specific resource allocation may improve efficiency and equity in long-term care systems. High-prevalence and high-risk clusters, such as the rapid full-dependency decline cluster (Cluster #12), may warrant targeted investment in end-of-life care pathways, advance care planning education for staff and families, and staffing models that prioritize supportive care [46]. Conversely, clusters with longer stable trajectories (e.g., Clusters #1, #2, and #5) may benefit from sustained investment in preventive care to delay functional decline. Finally, these findings support the development of cluster-informed clinical decision-support tools and quality metrics in long-term care. For example, facilities could be benchmarked on the proportion of residents in high-risk clusters who receive timely palliative referral, or on the rate of transitions from milder to severe disability trajectories as a potential indicator of care quality.

Overall, by moving beyond a one-size-fits-all approach and tailoring interventions, prognostication, and resource use to the 13 identified trajectory phenotypes, healthcare providers and policymakers may enhance personalization, optimize outcomes, reduce avoidable functional decline, and improve the efficiency and value of long-term care delivery.

### Limitations and future directions

While our study offers valuable methodological and empirical insights, several limitations should be noted. First, the empirical analysis is based on VA CLC residents, a group that might not fully represent all older adults, especially those in community nursing homes. Prior comparative studies indicate that VA CLC residents are primarily male, include a higher proportion of individuals under age 65, and often present with distinct clinical profiles, such as higher rates of trauma-related conditions, mental health comorbidities, and service-related disabilities. These characteristics can lead to greater baseline functional impairment, particularly among younger residents, and more heterogeneous ADL transition patterns in this setting. Meanwhile, older VA residents show many similarities to residents in non-VA nursing homes regarding functional status and impairment patterns, supporting the applicability of our findings to broader populations with functional impairment in nursing homes [47–49].

Second, although the hybrid clustering framework is population-agnostic, computationally scalable, and designed to uncover interpretable patterns in high-dimensional longitudinal data, empirical trajectory structures may vary in cohorts with different demographic compositions, care pathways, or assessment protocols. Future validation in diverse populations, including community-dwelling older adults and non-VA long-term care systems, and integration of additional domains (e.g., cognitive status, social support, multimorbidity) would further extend the approach.

While the specific trajectory phenotypes identified in this study reflect the characteristics of the VA CLC population and therefore may not generalize directly to other settings, the primary contribution of this work is methodological. The proposed framework, which integrates spell-based sequence construction for irregular observations, optimal-matching-based dissimilarity measures, and scalable quality-guided clustering procedures, is designed to address common challenges in large-scale longitudinal categorical data. Future work should evaluate the reproducibility of the identified cluster structures in external cohorts and assess sensitivity to key modeling choices.

Ultimately, given the highly skewed nature of the empirical state distribution, we focused our analysis on the 25 most frequently occurring states, which together comprised roughly 86% of all observed assessments. This decision mitigated sparsity and reduced computational demands while preserving the dominant recurring disability patterns used to define trajectory clusters. However, rare state combinations were not treated as distinct trajectory-defining states, and therefore, highly uncommon disability patterns may not be fully represented in the resulting trajectories. Consequently, the identified clusters should be interpreted as reflecting the major disability pathways observed in the population rather than exceptionally rare functional profiles.

## Conclusion

This work utilized a clustering approach to analyze and explore various groups of trajectories in detail. To address the computational challenges and limitations posed by existing SA tools, we developed a novel method that combines two clustering techniques based on Markov models and distance-based strategies. Through this approach, we identified 13 distinct clusters with unique characteristics, offering valuable insights into trajectories of functional loss and recovery. These insights have significant implications for personalized healthcare, enabling clinicians to predict functional decline and effectively tailor interventions. Furthermore, identifying clusters with unique care needs allows healthcare organizations to allocate resources more efficiently, potentially reducing costs and improving overall patient outcomes. Our work highlights the importance of adopting a cluster-based approach in analyzing patient pathways to enhance decision-making in healthcare settings.

## Supporting information

S1 Appendix OMspell distance details. (PDF)

S2 Appendix Complementary Tables. (PDF)

S3 Appendix Complementary Figures. (PDF)

S4 Appendix Additional Methodological Details. (PDF)
