# Supplementary material for: A clustering-based trajectory analytics of functional loss and recovery among older adults
Source: PLoS One. 2026 May 27;21(5):e0342424. doi: 10.1371/journal.pone.0342424 (PMC13215608; doi:10.1371/journal.pone.0342424)
Supplement: S1 Appendix — (PDF) [file pone.0342424.s001.pdf]

S1 Appendix. OMspell distance details

OMspell is a variant of the Optimal Matching (OM) distance that focuses specifically on the distances between *spells*, i.e., consecutive sequences of the same state. In OMspell, a spell is defined as a_t, representing state a with a duration of t time units, and is treated as a unique state. This formulation significantly increases the total number of distinct states in the analysis. To address the resulting growth in the number of parameters, [1] proposed a specialized cost structure for insertions, deletions, and substitutions, as outlined in Equation (S2). Their approach introduces a parameter δ that captures the cost associated with expanding or compressing sequences. Incorporating δ helps constrain the number of parameters required in the distance computation.

$$co_I(a_t) = co_I(a) + \delta(t-1) \tag{S1}$$

$$co_S(a_{t_1}, b_{t_2}) = \begin{cases} \delta|t_1 - t_2| & \text{if } a = b \\ co_S(a,b) + \delta(t_1 + t_2 - 2) & \text{otherwise} \end{cases} \tag{S2}$$

References

[1] Matthias Studer and Gilbert Ritschard. What matters in differences between life trajectories: a comparative review of sequence dissimilarity measures. *J R Stat Soc Ser A Stat Soc*, 179(2):481–511, 2016.
