# Supplementary material for: A clustering-based trajectory analytics of functional loss and recovery among older adults
Source: PLoS One. 2026 May 27;21(5):e0342424. doi: 10.1371/journal.pone.0342424 (PMC13215608; doi:10.1371/journal.pone.0342424)
Supplement: S2 Appendix — (PDF) [file pone.0342424.s002.pdf]

S2 Appendix. Complementary Tables

Table S1 provides complementary information on the 25 most common states, including their codes and associated severity levels.

Table S1: 25 most prevalent ADL states

State	Name	Severity
0	O	0
4	G	1
16	B	1
20	G B	2
32	W	1
48	B W	2
52	G B W	3
60	G T B W	4
84	G B D	3
92	G T B D	4
116	G B W D	4
124	G T B W D	5
126	S G T B W D	6
127	F S G T B W D	7
252	G T B W D L	6
254	S G T B W D L	7
255	F S G T B W D L	8
276	G B U	3
348	G T B D U	5
380	G T B W D U	6
476	G T B D L U	6
508	G T B W D L U	7
509	F G T B W D L U	8
510	S G T B W D L U	8
511	All	9

Table S2 compares the performance of the selection methods (density, centrality, frequency, likelihood) in identifying representative sets for each cluster based on their coverage, number of representatives, and gain.

Table S2: Comparison of representative sets using different criteria

**Cluster #**	**Method**	**Coverage (%)**	**# of Rep.**	**Gain**
1	Density	**77.32**	23	10.88
	Centrality	75.04	16	6.21
	Frequency	75.60	**3**	6.62
	**Likelihood**	75.75	**4**	**12.05**
2	Density	**78.439**	**6**	12.18
	Centrality	75.36	**4**	8.65
	Frequency	75.15	15	12.28
	**Likelihood**	77.40	**3**	**14.11**
4	Density	75.07	13	10.60
	Centrality	75.19	13	10.98
	Frequency	75.14	**4**	13.81
	**Likelihood**	**77.30**	**4**	**17.40**
5	Density	75.00	**6**	-8.59
	**Centrality**	**78.30**	**2**	**2.24**
	Frequency	77.80	**2**	1.41
	Likelihood	76.40	**2**	-1.51
6	Density	77.39	14	8.97
	Centrality	75.61	**4**	6.06
	**Frequency**	**77.68**	**3**	**12.59**
	Likelihood	77.20	**3**	12.28
8	Density	**77.50**	**1**	-13.00
	**Centrality**	**77.50**	**1**	**-11.80**
	Frequency	77.40	**1**	-13.40
	Likelihood	75.60	**1**	-23.90
10	Density	**80.90**	**1**	-19.60
	**Centrality**	80.70	**1**	**-12.10**
	Frequency	80.80	**1**	-13.90
	Likelihood	**80.90**	**1**	-17.40
11	Density	**71.38**	**10**	10.32
	Centrality	68.76	**10**	5.49
	Frequency	63.86	**10**	-10.01
	**Likelihood**	71.19	**10**	**10.47**
12	Density	**91.60**	**1**	-4.96
	**Centrality**	**91.60**	**1**	**-4.03**
	Frequency	91.30	**1**	-8.11
	Likelihood	91.50	**1**	-5.39
13	Density	75.30	**1**	-24.50
	**Centrality**	**79.90**	**2**	**-0.27**
	Frequency	75.10	**1**	-22.10
	Likelihood	75.00	**1**	-22.60
14	Density	**80.50**	**1**	-15.40
	**Centrality**	80.30	**1**	**-14.60**
	Frequency	80.20	**1**	**-14.60**
	Likelihood	75.90	**1**	-40.40

Continued on the next page

Continued from the previous page

Cluster #	Method	Coverage (%)	# of Rep.	Gain
15	Density	65.92	**10**	7.02
	Centrality	62.24	**10**	0.353
	Frequency	60.00	**10**	-1.41
	**Likelihood**	**67.73**	10	**9.91**
18	Density	76.60	**1**	-18.30
	Centrality	76.50	**1**	-18.30
	Frequency	**77.70**	**2**	-10.41
	**Likelihood**	**77.70**	2	**-10.08**

Table S3: Technical configuration of the trajectory clustering procedure

**Component**	**Specification**
Software Environment	R (version 4.1)
Sequence Analysis Package	TraMineR (v2.2-7)
Clustering Framework	Two-stage clustering: initial EM-based partitioning followed by refinement using PAM
Sequence Distance Metric	OMspell (Optimal Matching for spell/state-duration sequences)
Substitution Cost Matrix	Feature-based cost matrix derived from ADL severity scores
Insertion/Deletion (INDEL) Cost	Automatically defined as half of the maximum substitution cost
Expansion Cost	0.1
Initial Partitioning Method	Expectation–Maximization (EM) clustering based on transition matrices
Initial Number of Clusters	$k = 4$
Refinement Algorithm	Partitioning Around Medoids (PAM) implemented via wcKMedoids
Initial Medoid Selection	Average linkage (UPGMA) hierarchical clustering
Final Number of Clusters	13
Primary Cluster Quality Metric	Weighted Average Silhouette Width (ASWw)

Table S4 presents the demographic distribution of male and female residents across the 13 distinct functional trajectory clusters.

Table S4: Distribution of clusters by sex

	Male		Female	
Cluster	Count	%	Count	%
1	6813	6.39%	215	6.49%
12	55748	52.27%	1666	50.32%
2	1783	1.67%	41	1.24%
4	3732	3.50%	103	3.11%
5	2836	2.66%	125	3.78%
6	4813	4.51%	167	5.04%
8	3665	3.44%	99	2.99%
10	7657	7.18%	230	6.95%
11	2995	2.81%	141	4.26%
13	8769	8.22%	255	7.70%
14	3437	3.22%	118	3.56%
15	1729	1.62%	95	2.87%
18	2668	2.50%	56	1.69%

Table S5: Data dictionary describing the variables, coding scheme, and dataset structure

Variable Name	Description	Type	Notes
ID	Unique patient identifier	Integer / Character	One identifier per individual
age	Age at assessment (years)	Numeric	Age at time of assessment
sex	Biological sex	Categorical	e.g., 1 = Female, 0 = Male
nass	Total number of assessments per patient	Integer	Calculated across longitudinal records
followed	Total follow-up duration	Integer	Time from first to last recorded assessment (days)
dayfirst	Day of the first assessment	Numeric (days)	Used to compute follow-up duration
daylast	Day of the last assessment	Numeric (days)	Used to compute follow-up duration
Ueat	Eating (feeding)	Binary	Functional status for eating
Usit	Transferring/sitting	Binary	Functional status for sitting/transferring
Ugroom	Grooming	Binary	Functional status for grooming
Utoil	Toileting	Binary	Functional status for toilet use
Ubath	Bathing	Binary	Functional status for bathing
Uwalk	Walking	Binary	Functional status for walking
Udress	Dressing	Binary	Functional status for dressing
Ubowel	Bowel continence	Binary	Bowel control status
Uurin	Urinary continence	Binary	Urinary control status
live	Mortality flag	Binary	0 = alive; 1 = deceased
assn	Assessment number/sequence index	Integer	Chronological order of assessment per patient
code	Encoded composite ADL state	Integer	Represents combined multi-domain ADL functional state
