# Supplementary material for: A clustering-based trajectory analytics of functional loss and recovery among older adults
Source: PLoS One. 2026 May 27;21(5):e0342424. doi: 10.1371/journal.pone.0342424 (PMC13215608; doi:10.1371/journal.pone.0342424)
Supplement: S3 Appendix — (PDF) [file pone.0342424.s003.pdf]

S3 Appendix. Complementary Figures

This appendix presents supplementary figures that provide additional visual context to support the main sections of the document. Figure S3 includes a legend illustrating the various states and their corresponding colours, which appear consistently throughout the plots in the main text. Figures S4–S7 show the initial sub-clusters derived from four primary clusters, while Figure S8 highlights changes in the state distribution of the remaining clusters. Figures S9 display the relative frequency plots of the final clusters, representing the medoids of 250 equally sized, sorted trajectory groups per cluster. These plots provide a clear view of the distinct patterns of functional decline and recovery across disability states. Note that although the final solution includes 13 clusters, their numbering is non-sequential due to merges during the optimization process. For instance, Figures S9 show clusters labelled #1, #2, and #4, indicating that cluster #3 was merged. This should be kept in mind when interpreting the visualizations. Finally, Figures S10 illustrate representative trajectories for each cluster. The representative trajectory for cluster #12 is (52,2), and its corresponding plot has been zoomed in for clarity.

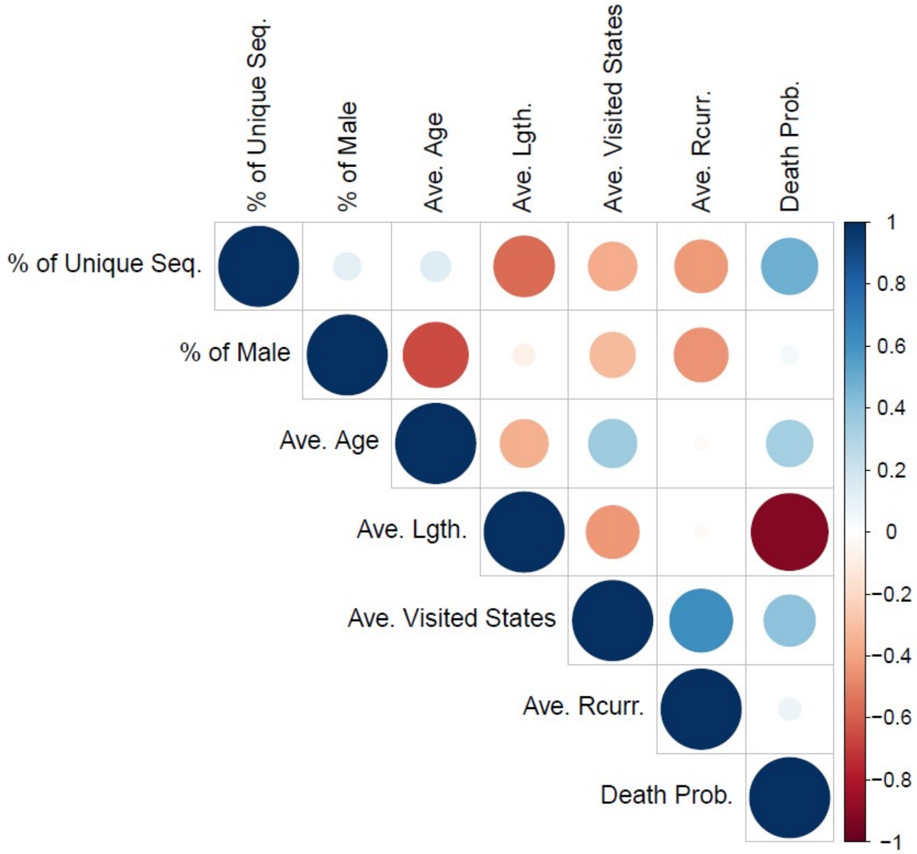

Figure S1: Correlation of different characteristics of the cluster profiles

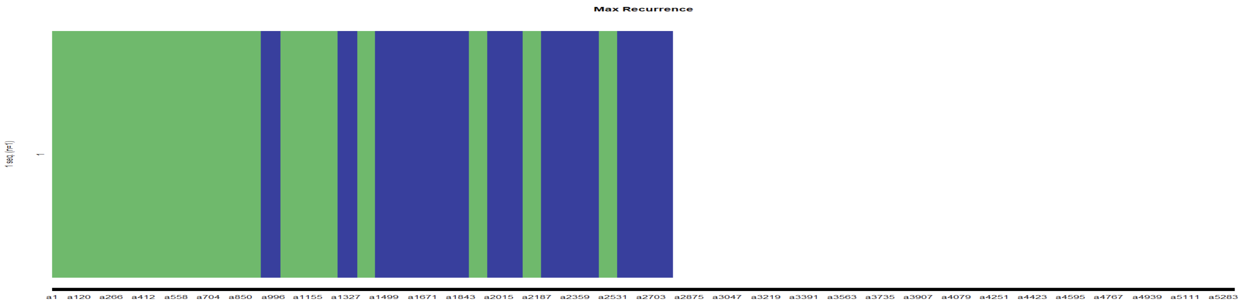

Figure S2: Example of the trajectories in the dataset

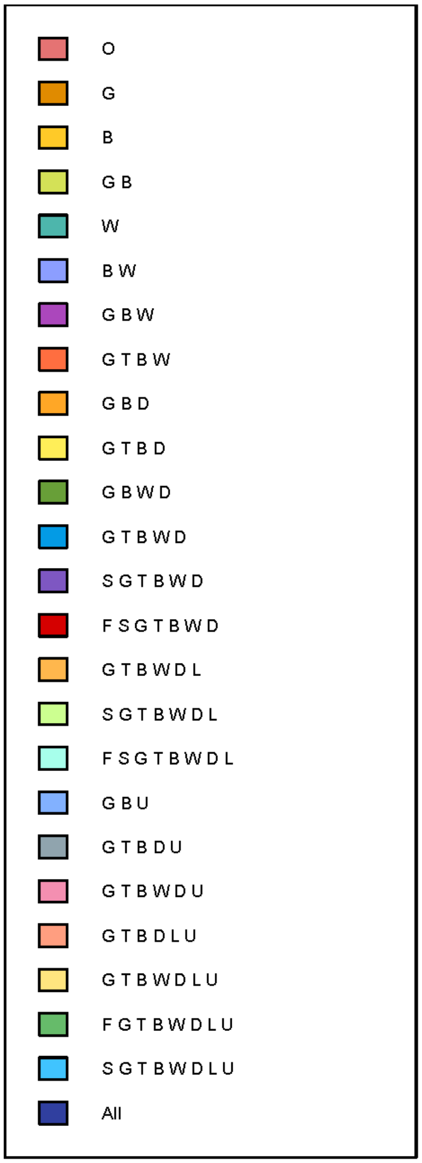

Figure S3: Legend for the states (disability combinations)

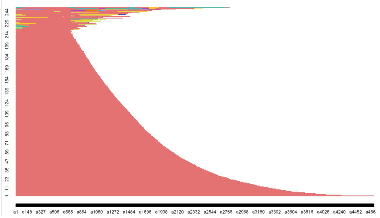

(a) 1st sub-cluster of cluster #1: Group medoids

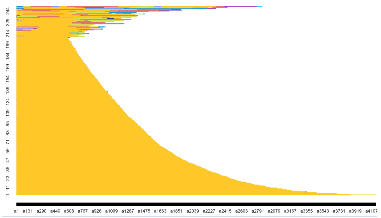

(b) 2nd sub-cluster of cluster #1: Group medoids

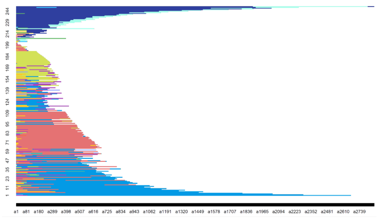

(c) 3rd sub-cluster of cluster #1: Group medoids

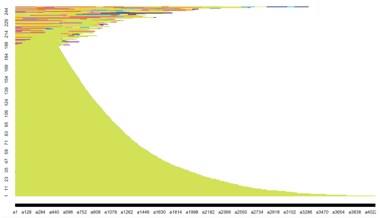

(d) 4th sub-cluster of cluster #1: Group medoids

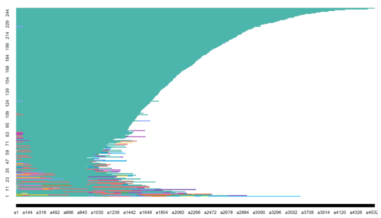

(e) 5th sub-cluster of cluster #1: Group medoids

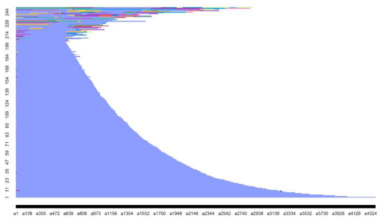

(f) 6th sub-cluster of cluster #1: Group medoids

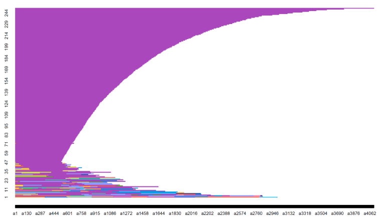

(g) 7th sub-cluster of cluster #1: Group medoids

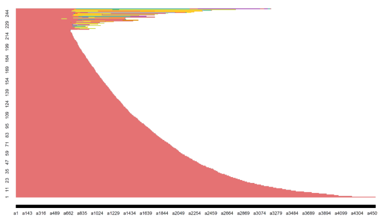

(h) 8th sub-cluster of cluster #1: Group medoids

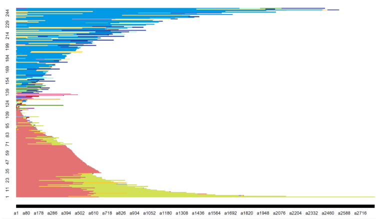

(i) 9th sub-cluster of cluster #1: Group medoids

Figure S4: Cluster #1 generating nine sub-clusters: Relative frequency plots

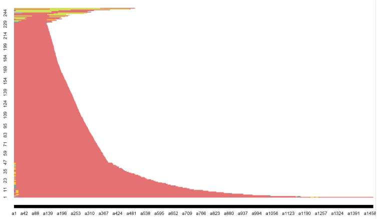

(a) 1st sub-cluster of cluster #2: Group medoids

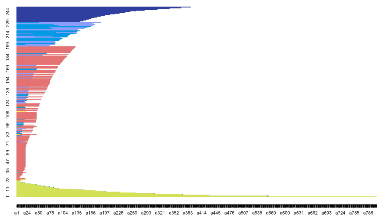

(b) 2nd sub-cluster of cluster #2: Group medoids

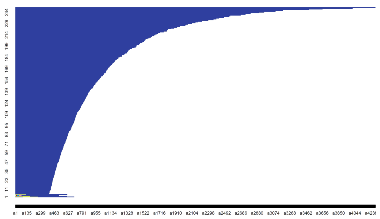

(c) 3rd sub-cluster of cluster #2: Group medoids

Figure S5: Cluster #2 generating three sub-clusters: Relative frequency plots

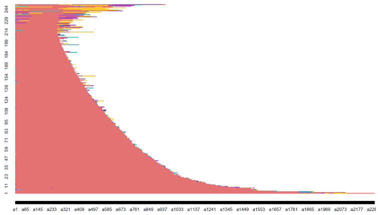

(a) 1st sub-cluster of cluster #3: Group medoids

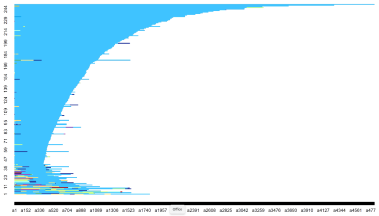

(b) 2nd sub-cluster of cluster #3: Group medoids

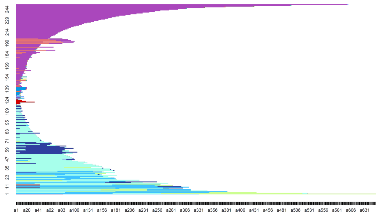

(c) 3rd sub-cluster of cluster #3: Group medoids

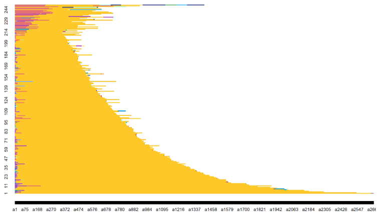

(d) 4th sub-cluster of cluster #3: Group medoids

Figure S6: Cluster #3 generating four sub-clusters: Relative frequency plots

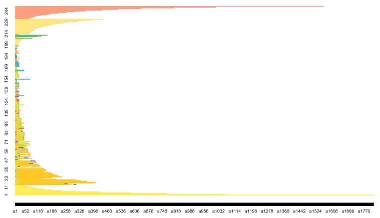

(a) 1st sub-cluster of cluster #4: Group medoids

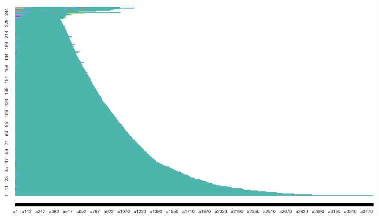

(b) 2nd sub-cluster of cluster #4: Group medoids

Figure S7: Cluster #4 generating two sub-clusters: Relative frequency plots

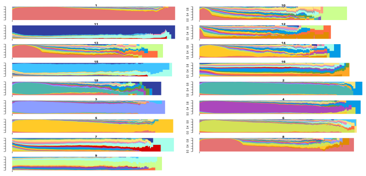

(a) Cluster #17 merged into others

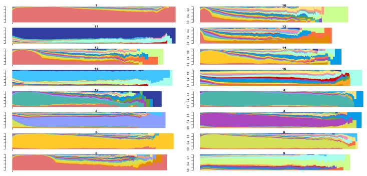

(b) Cluster #7 merged into others

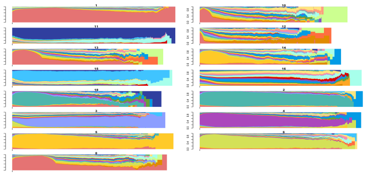

(c) Cluster #9 merged into others

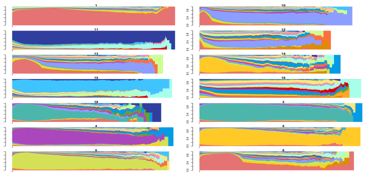

(d) Cluster #3 merged into others

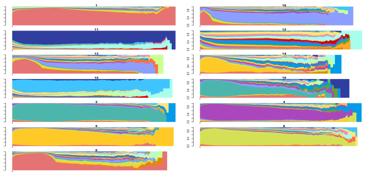

(e) Cluster #16 merged into others

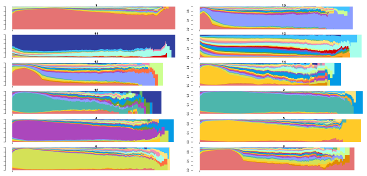

(f) Cluster #15 merged into others

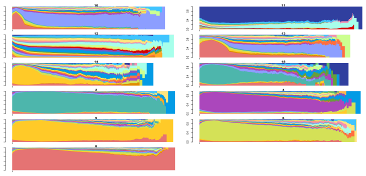

(g) Cluster #1 merged into others

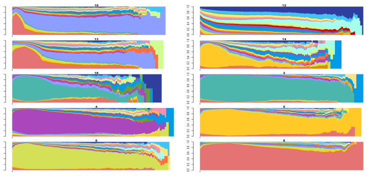

(h) Cluster #11 merged into others

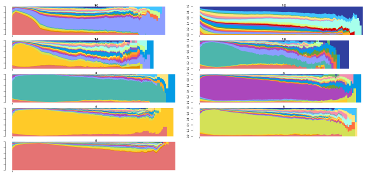

(i) Cluster #13 merged into others

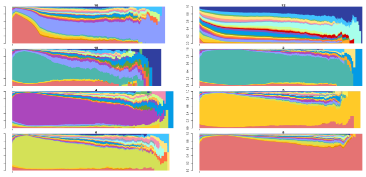

(j) Cluster #14 merged into others

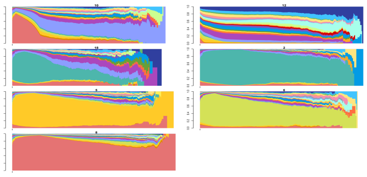

(k) Cluster #4 merged into others

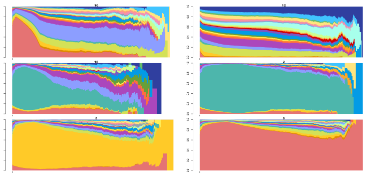

(l) Cluster #6 merged into others

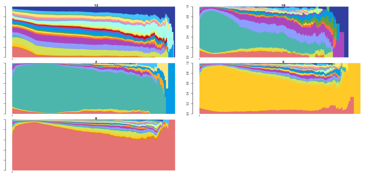

(m) Cluster #10 merged into others

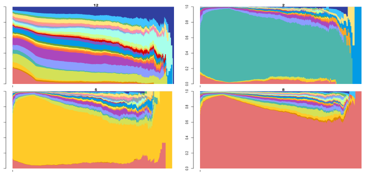

(n) Cluster #18 merged into others

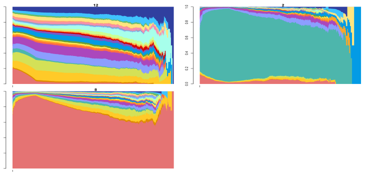

(o) Cluster #5 merged into others

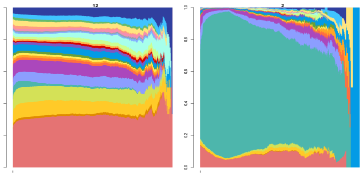

(p) Cluster #8 merged into others

Figure S8: Clusters merging into one another during the quality assessment step

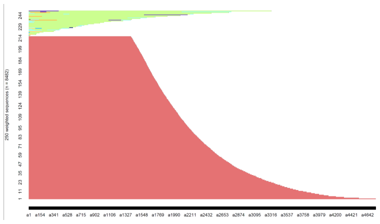

(a) Final cluster #1: Group medoids

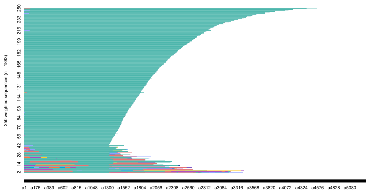

(b) Final cluster #2: Group medoids

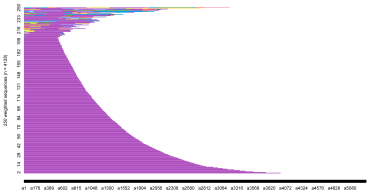

(c) Final cluster #4: Group medoids

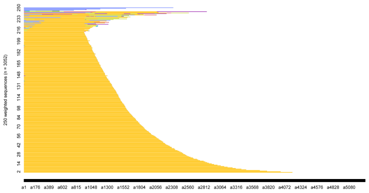

(d) Final cluster #5: Group medoids

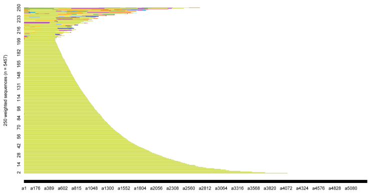

(e) Final cluster #6: Group medoids

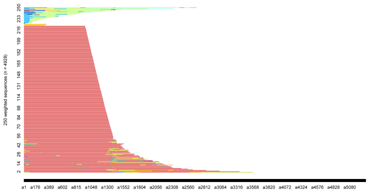

(f) Final cluster #8: Group medoids

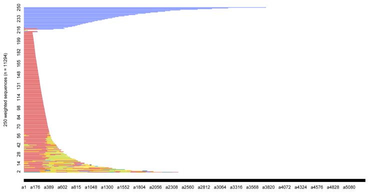

(g) Final cluster #10: Group medoids

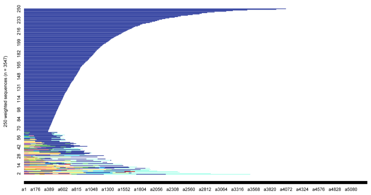

(h) Final cluster #11: Group medoids

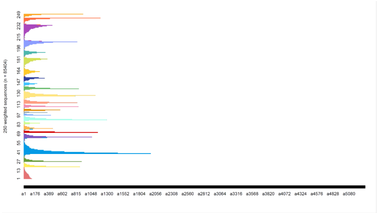

(i) Final cluster #12: Group medoids

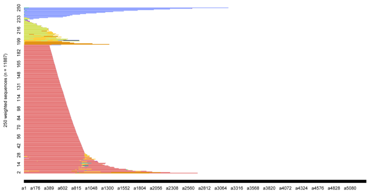

(j) Final cluster #13: Group medoids

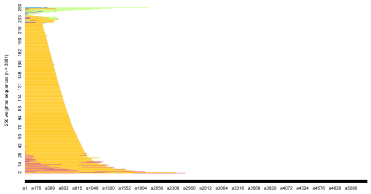

(k) Final cluster #14: Group medoids

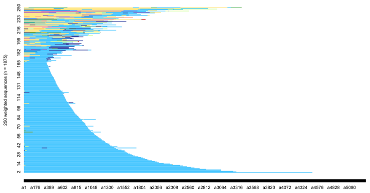

(l) Final cluster #15: Group medoids

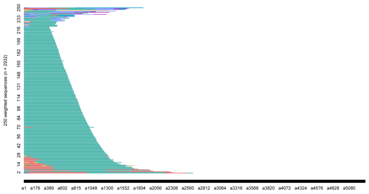

(m) Final cluster #18

Figure S9: 13 Final Clusters: Relative frequency plots

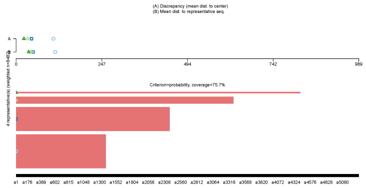

(a) Representative trajectory of cluster #1

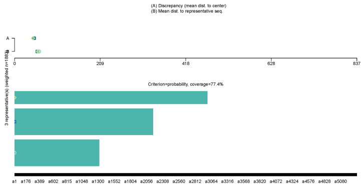

(b) Representative trajectory of cluster #2

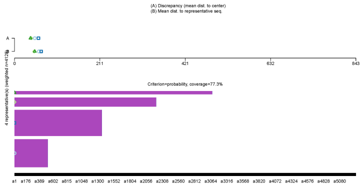

(c) Representative trajectory of cluster #4

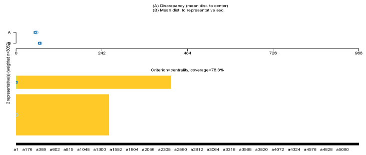

(d) Representative trajectory of cluster #5

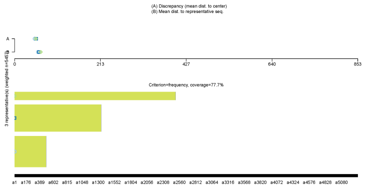

(e) Representative trajectory of cluster #6

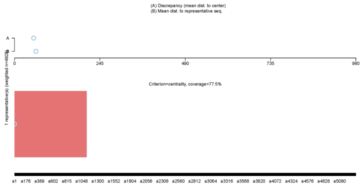

(f) Representative trajectory of cluster #8

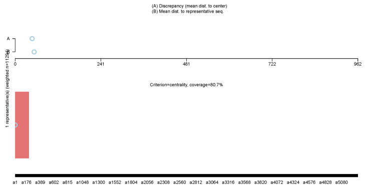

(g) Representative trajectory of cluster #10

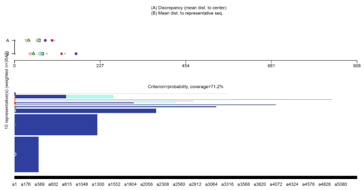

(h) Representative trajectory of cluster #11

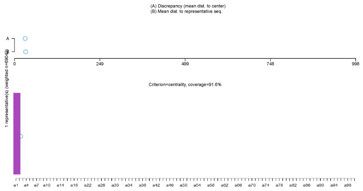

(i) Representative trajectory of cluster #12

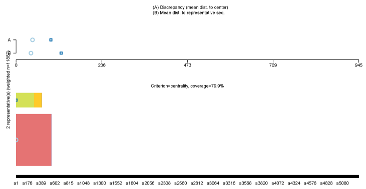

(j) Representative trajectory of cluster #13

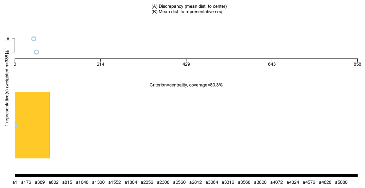

(k) Representative trajectory of cluster #14

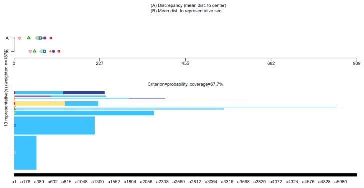

(l) Representative trajectory of cluster #15

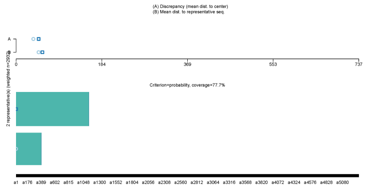

(m) Representative trajectory of cluster #18

Figure S10: Representative set of final clusters

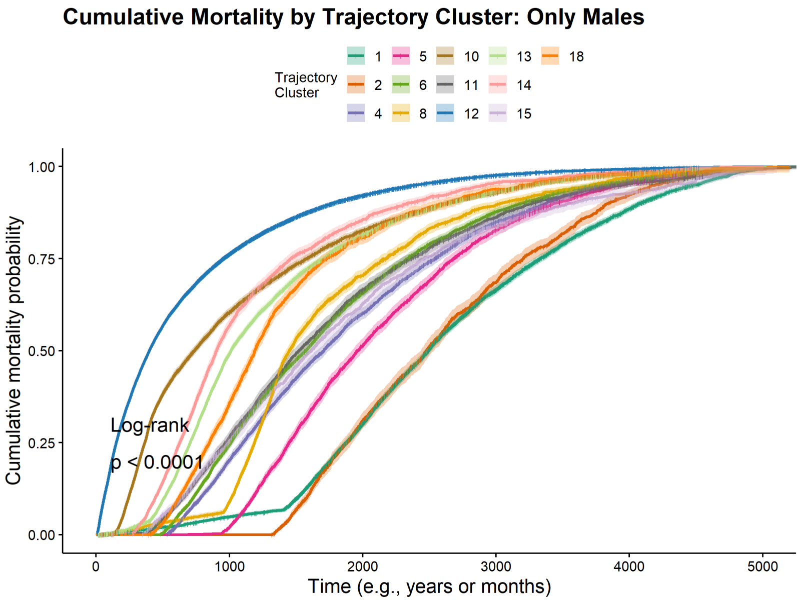

Figure S11: Probability of death over time for different clusters, Males only

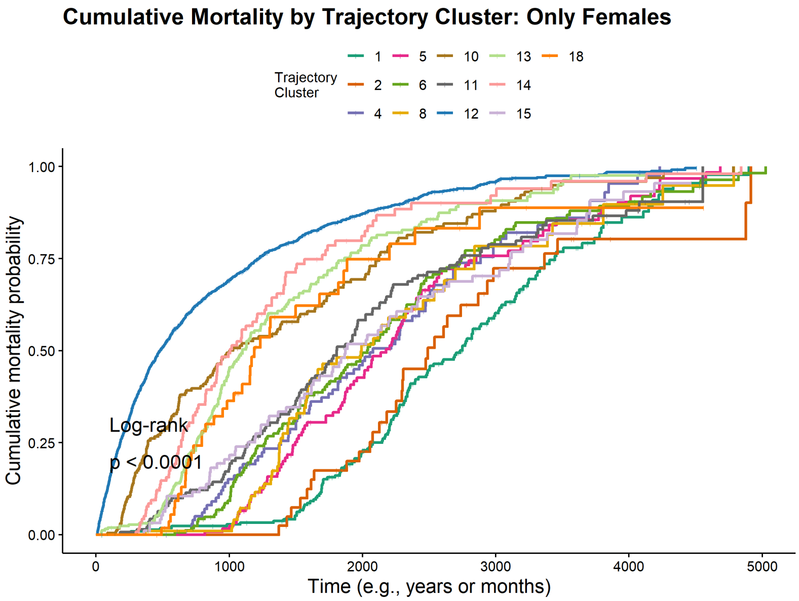

Figure S12: Probability of death over time for different clusters, Females only
