# Supplementary material for: A clustering-based trajectory analytics of functional loss and recovery among older adults
Source: PLoS One. 2026 May 27;21(5):e0342424. doi: 10.1371/journal.pone.0342424 (PMC13215608; doi:10.1371/journal.pone.0342424)
Supplement: S4 Appendix — (PDF) [file pone.0342424.s004.pdf]

S4 Appendix. Additional Methodological Details

In the second phase of our clustering approach, we use pair-wise dissimilarities to further group the four primary clusters into more refined clusters. There are various distance-based clustering approaches, and Studer (2013)[10] categorized them based on two logics: hierarchical clustering methods, and algorithms requiring a predefined number of clusters (k), such as the Partitioning Around Medoids (PAM) algorithm. Studer (2013) suggested combining the two distance-based approaches to overcome each method's limitations while leveraging both methods' strengths. To elaborate, hierarchical clustering can occasionally become stuck in local optima. Additionally, PAM relies on and is sensitive to the randomness of the initial choices of k medoids. The combined approach begins by applying hierarchical clustering to the trajectories, which helps obtain an initial set of medoids for the subsequent PAM algorithm. This approach reduces randomness in the PAM algorithm and enhances the clustering outcome.

Phase 2

Step 2.1: Hierarchical Clustering: After forming primary clusters using Markov chains, we applied hierarchical clustering—a distance-based technique—to these clusters. Hierarchical clustering methods can be broadly categorized into two types: agglomerative and divisive. We chose the agglomerative approach, which is the most widely used method in hierarchical clustering. This technique begins by treating each observation as an individual cluster and then iteratively merging the closest clusters until all data is encompassed within a single cluster [7]. Various evaluation criteria exist to determine which two clusters should be merged or split. In a comparative study conducted by Roux (2018), nine different criteria were examined [9]. The study's findings indicated that within the context of the agglomerative algorithm, the average link criterion demonstrated one of the best performances, striking a favorable balance between clustering quality and computational efficiency. The average link criterion calculates the mean distance between pairs of observations from different clusters [9]. Based on these insights, we concluded that the average link criterion is an excellent choice for our analytics, as it offers a harmonious blend of quality and computational speed.

Step 2.2: Partitioning Around Medoids (PAM) Clustering: The clusters obtained from hierarchical clustering are further refined using the PAM algorithm, which starts with the medoids identified in the hierarchical step. This stage enhances clustering by iteratively optimizing the choice of medoids to minimize dissimilarity within the clusters.

The PAM algorithm, developed by Kaufman and Rousseeuw (1990) [6], aims to identify k objects as representatives of k clusters, called medoids, the most central objects selected from the actual data. It seeks to minimize the overall dissimilarity between these representatives and the other objects in their respective clusters. This algorithm comprises two stages: "BUILD" and "SWAP." In the first stage, it aims to identify the initial set of k clusters by selecting the initial medoids. The second stage enhances the clustering solution. During this stage, the algorithm iteratively swaps the current medoids with other objects within the same cluster, evaluates the resulting dissimilarity, and selects new medoids with the highest potential to minimize distances. This iterative process

continues until no further improvements can be achieved [10]. Effective application of these distance-based clustering algorithms requires careful consideration of dissimilarity measures, substitution and indel costs, and the selection of an optimal number of clusters. These topics are discussed in the following three subsections.

Measuring Dissimilarities: The first requirement in performing distance-based clustering is to measure the differences among sequences. In the context of SA, these differences, referred to as dissimilarities, can be calculated in several ways. The three main types are: distribution-based distances, which compare overall patterns; attribute-based distances, which count the characteristics shared by sequences; and edit distances, such as Optimal Matching (OM), which measure the effort required to transform one sequence into another [11]. Sequence analysts primarily use the OM algorithm and its variants to evaluate distances between sequences, as it is particularly effective at identifying similarities and differences in sequence patterns. The standard OM algorithm relies on three essential operations: replacement, insertion, and deletion. Each operation is associated with a cost, and the main goal of the OM algorithm is to convert a primary sequence into another target sequence with the minimum overall cost. By utilizing dynamic programming, the algorithm determines the minimum cost and returns it as the distance between the two provided sequences [1].

According to a thorough review of sequence dissimilarity measures, capturing all three dimensions of sequences, i.e., sequencing, timing, and duration within a single measure, remains a complex challenge in SA [11]. Traditional methods often fall short in addressing all three dimensions. Recent advancements, such as OMspell, aim to enhance dissimilarity assessment by focusing on distances between spells—defined as continuous periods spent in the same state—providing a more nuanced alternative to previous approaches [11]. OMspell, unlike the standard OM algorithm, demonstrates high sensitivity to sequencing, making it a promising option for analyzing the order of states in functional loss and recovery trajectories. Additionally, the algorithm is responsive to the duration of events, further enhancing OM's ability to examine disease progression patterns. One advantage of OMspell is its capacity to control the sensitivity to all three dimensions: Adjust parameters to control sequencing, timing, and duration. For instance, by lowering the expansion cost parameter, the measure becomes better aligned with the event order, enabling a thorough analysis of sequence patterns. The detailed explanation of OMspell can be found in Appendix S1.

Substitution and indel costs: As mentioned previously, the OM algorithm calculates the distance between two sequences using three primary functions: replacement (or substitution), insertion, and deletion. Typically, an equal cost is assigned to both insertion and deletion operations; this common cost is referred to as the "indel" cost. Several strategies can be employed to define the substitution and indel costs. For substitution cost, there are three distinct approaches: theoretically derived, based on state attributes, and data-driven (such as transition-based) [11]. In our study, we determine substitution costs based on the attributes of the states. This approach allows us to assign costs that reflect the level of similarity between states and meaningfully evaluate their closeness. Specifically, we consider the severity levels of the disability combinations to establish the substitution costs. By incorporating state attributes into the calculation of substitution costs, we can capture the true nature of the states within the sequences, making our analysis more practical and enhancing its accuracy and relevance. For indel costs, two groups of approaches can be utilized: single cost and state-dependent costs. In the context of state-dependent indel costs, Studer and Ritschard (2016) proposed various methods [11].

One method involves employing a monotone function based on the inverse of state frequency, which considers the relative occurrence of each state. Another approach is to take into account the inverse of the mean time spent in each state, capturing the duration aspect of the states. Additionally, the time not spent in the states can also be regarded as a measure of indel costs. In

our study, we opted for the default single-cost approach for the sake of simplicity. This approach assigns a single cost value for both insertions and deletions. The specific equation used to calculate the indel cost in our analysis is provided in Equation S1 [11]. This simplification allows us to streamline the calculation process and facilitate easier interpretation of the results.

$$\frac{co_S^{max}}{2} \leq co_I \leq A \times \frac{co_S^{max}}{2} \tag{S1}$$

where co_S^{max} is the maximum substitution cost, co_I is the indel cost, and A is the maximum sequence length.

Determination of Optimal Number of Clusters: As the PAM algorithm refines clustering through the BUILD and SWAP stages, determining the most effective number of clusters to finalize the structure becomes essential. This determination involves a rigorous evaluation of clustering quality. To find the optimal number of clusters for the PAM algorithm, we can experiment with different values of k and select the best one according to a quality metric. In our study, we use the average silhouette width, weighted (ASWw) measure to assess the quality of the clustering solutions. In this method, the ASW score serves as an indicator of the proximity or separation of the clusters. Specifically, the silhouette score assesses whether an object is correctly assigned to its respective cluster or if it is closer to an adjacent cluster. To achieve this, it calculates the average distance between the observation and other objects within its own cluster, as well as the average distance between the observation and other objects in the nearest adjacent cluster. To evaluate the overall clustering quality, the silhouette score is computed for each observation, and the mean value is determined. This mean score, known as ASW, ranges from -1 to 1, where a negative value indicates poor clustering and the misclassification of some objects. A positive value, on the other hand, indicates a strong clustering structure, signifying that most objects are well-assigned to their respective clusters. Therefore, ASW serves as a reliable measure of overall clustering performance, particularly in the context of our study [8]. The weighted version of ASW, referred to as ASWw, is proposed by Studer (2013) to consider the weights assigned to each observation [10]. By incorporating the weights, ASWw provides a comprehensive assessment that takes into account the impact of each observation on the clustering results. In summary, the ASWw metric delivers a quantitative evaluation of the quality and cohesion of the clusters. It enables comparisons between different clustering solutions and helps select the most suitable number of clusters for our analysis. The goal is to determine the value of k that results in the highest weighted average silhouette width, signifying the optimal clustering solution.

Phase 3

Step 3.1: Global Reassignment By employing a combined distance-based algorithm on the primary clusters obtained from Markov chains in the first phase, we generate multiple sub-clusters. These sub-clusters are then used to compute a smaller distance matrix that includes all observations. Utilizing these distances, the trajectories are subsequently reassigned to the closest medoid based on proximity. This strategy leverages the power of distance-based algorithms without a computational burden, resulting in an initial clustering solution. However, a critical aspect to consider is whether this clustering solution exhibits high quality. Therefore, the next step in the process focuses on evaluating the quality and determining the optimal clustering setting.

Step 3.2: Optimal Clustering Solution

Our quality assessment of the clustering settings consists of an iterative process with the following steps:

1. Use the ASWw metric to measure the quality of each cluster.

2. Identify the cluster with the lowest quality, indicated by the smallest value of ASWw.

3. Merge the selected cluster with other clusters by assigning each trajectory to its closest neighboring cluster.

4. Recompute the medoids of clusters that have received new trajectories.

5. Repeat steps 1-4 until only two clusters remain.

An important question is which clustering setting yields the optimal solution. To assess clustering quality, we utilized five commonly employed metrics [10]. The first, known as "Hubert's Somers' D" (HGSD), evaluates quality by examining the association between two distance matrices [5]. This metric measures the capacity to regenerate the original distance matrix based on the number of concordant and discordant objects. The second metric, referred to as "Hubert's C" index (HC), compares the current clustering solution to the best theoretically achievable solution, measuring its performance relative to the ideal clustering scenario. The third metric, the "Calinski-Harabasz" index (CH), employs the F-statistic from ANOVA to assess the compactness of the clusters through the sum of squared distances within each cluster [2]. Additionally, we consider the pseudo-R^2 metric, which measures the variance explained by the clustering solution [12]. Lastly, we utilize the ASWw metric to evaluate the clustering quality. It is essential to note that, generally, all these metrics should be maximized to achieve the highest quality in clustering, with the exception of the HC metric which should be minimized. Finally, the optimal solution is selected through the simultaneous evaluation of all quality metrics, ensuring a comprehensive and balanced assessment of clustering performance.

Representation Learning

After determining the optimal clustering structure based on the aforementioned metrics, we analyzed and explored different trajectory profiles within the dataset. Furthermore, we predicted the likelihood of death within each final cluster to understand its association with ADL trajectory clusters. Several criteria have been proposed to identify representative sets in SA, including neighborhood density, centrality, frequency, and likelihood [4, 3]. The neighborhood density method defines a neighborhood radius and counts the number of objects within that radius. The sequences exhibiting the highest densities, which indicate greater coverage of neighboring objects, are then selected to illustrate representatives [4]. In the centrality criterion that follows the classical logic of medoids, the object with the minimum sum of within-cluster distances is chosen as the representative of the cluster. This approach aims to find the observation that is most centrally located within its cluster. The frequency criterion involves sorting the sequences based on their frequency and selecting representatives based on their occurrence in the dataset. This approach prioritizes the most frequently observed sequences as representatives. The likelihood criterion takes a different approach by using statistical modeling to define representative sets. It computes the product of the probabilities of states occurring at each time point. Typically, a Markov model is used to estimate the probability values, allowing for identifying representative sequences based on their likelihood of occurrence. By considering these criteria for selecting representative sets, we can achieve a deeper understanding of the data and identify typical trajectories that reflect the underlying patterns of functional loss and recovery within each cluster. Each criterion offers a different perspective and may be appropriate depending on the dataset and clusters' structures. To ensure a thorough evaluation, we applied the four criteria mentioned above to each cluster and selected the representative set that demonstrated the highest quality. Various measures can be employed to assess the quality of the representative sets, including mean distance, coverage, distance gain, and discrepancy [4, 3].

Among these measures, we focus on the coverage and the distance gain to select the optimal set of representatives for each cluster. The reason for choosing these two metrics is that, based on our observations, the discrepancy results in the same value for all four measures within the same cluster, making it unsuitable for comparison. Moreover, the mean distance criterion utilizes distances to the representatives, which are also used in the gain criterion. Our experimental results confirm a similar behavior between the mean distance and distance gain measures. The results of mean distance and discrepancy measures can be found in Appendix S2. Therefore, we utilized gain, which also provides a valuable comparison between the distances to the medoid and the representatives. To further elaborate, the objective of the gain measure is to determine whether the new representatives reduce the total within-cluster distances compared to the total distances to the medoid, as illustrated in Equation S2.

$$Q = \sum_{i}^{nr} \frac{DC_i - SD_i}{DC_i} \tag{S2}$$

Here, DC_i represents the distance of object x_i to to the cluster's center, while SD_i represents the distance of object x_i to the selected representative. For a group of representatives, the gain measure is computed for each one, and the average of all gain values across the representatives serves as an indicator of the overall quality of the representative set. This measure allows us to assess the effectiveness of the representatives in minimizing within-cluster distances. Finally, the concept of the coverage criterion differs from the others, as it counts the number of sequences that fall within a predefined distance and presents it as a percentage. In our analytics, we applied all four measures to obtain representative sets for each cluster and carefully evaluated the quality of the sets generated by each method using the coverage, number of representatives, and gain values.

References

[1] Andrew Abbott and Angela Tsay. Sequence Analysis and Optimal Matching Methods in Sociology: Review and Prospect. *Sociological Methods & Research*, 29(1):3–33, August 2000. Publisher: {SAGE} Publications Inc.

[2] T. Caliński and J Harabasz. A dendrite method for cluster analysis. *Communications in Statistics*, 3(1):1–27, January 1974. Publisher: Taylor & Francis _eprint: https://www.tandfonline.com/doi/pdf/10.1080/03610927408827101.

[3] Alexis Gabadinho and Gilbert Ritschard. Searching for typical life trajectories applied to childbirth histories. pages 287–312. January 2013.

[4] Alexis Gabadinho, Gilbert Ritschard, Matthias Studer, and Nicolas S. Müller. Extracting and Rendering Representative Sequences. In Ana Fred, Jan L. G. Dietz, Kecheng Liu, and Joaquim Filipe, editors, *Knowledge Discovery, Knowledge Engineering and Knowledge Management*, Communications in Computer and Information Science, pages 94–106, Berlin, Heidelberg, 2011. Springer.

[5] Lawrence Hubert and Phipps Arabie. Comparing partitions. *Journal of Classification*, 2(1):193–218, December 1985.

[6] Leonard Kaufman and Peter J. Rousseeuw. Partitioning Around Medoids (Program PAM). In *Finding Groups in Data*, pages 68–125. John Wiley & Sons, Ltd, 1990. Section: 2 _eprint: https://onlinelibrary.wiley.com/doi/pdf/10.1002/9780470316801.ch2.

[7] Glenn W. Milligan and Martha C. Cooper. Methodology Review: Clustering Methods. *Applied Psychological Measurement*, 11(4):329–354, December 1987. Publisher: SAGE Publications Inc.

[8] Peter J. Rousseeuw. Silhouettes: A graphical aid to the interpretation and validation of cluster analysis. *Journal of Computational and Applied Mathematics*, 20:53–65, November 1987.

[9] Maurice Roux. A Comparative Study of Divisive and Agglomerative Hierarchical Clustering Algorithms. *J Classif*, 35(2):345–366, July 2018.

[10] Matthias Studer. WeightedCluster Library Manual: A practical guide to creating typologies of trajectories in the social sciences with R. *LIVES*, January 2013.

[11] Matthias Studer and Gilbert Ritschard. What matters in differences between life trajectories: a comparative review of sequence dissimilarity measures. *J R Stat Soc Ser A Stat Soc*, 179(2):481–511, 2016.

[12] Matthias Studer, Gilbert Ritschard, Alexis Gabadinho, and Nicolas S. Müller. Discrepancy Analysis of State Sequences. *Sociological Methods & Research*, 40(3):471–510, August 2011. Publisher: SAGE Publications Inc.
